# CircIMMP2L promotes esophageal squamous cell carcinoma malignant progression via CtBP1 nuclear retention dependent epigenetic modification

**DOI:** 10.1002/ctm2.519

**Published:** 2021-09-26

**Authors:** Yingkuan Liang, Qixing Mao, Lin Wang, Wenjie Xia, Bing Chen, Hui Wang, Rutao Li, Lin Xu, Feng Jiang, Gaochao Dong

**Affiliations:** ^1^ Department of Thoracic Surgery Jiangsu Key Laboratory of Molecular and Translational Cancer Research, Jiangsu Cancer Hospital, Jiangsu Institute of Cancer Research The Affiliated Cancer Hospital of Nanjing Medical University Nanjing P.R. China; ^2^ Department of Thoracic Surgery the First Affiliated Hospital of Soochow University Suzhou P.R. China; ^3^ Department of Oncology, Department of Geriatric Lung Cancer Laboratory Geriatric Hospital of Nanjing Medical University Nanjing P.R. China

**Keywords:** circIMMP2L, circRNA, ESCC, CtBP1, HDAC1

## Abstract

**Background:**

Esophageal squamous cell carcinoma (ESCC) is one of the most aggressive cancers. The two major lethal causes of ESCC are diagnosis at an advanced stage and lymph node metastasis (LNM). Circular RNAs (circRNAs) play critical regulatory roles in cancer progression, though, largely through unclear mechanisms. However, the character of circRNAs in the malignant progression of ESCC remains unclear.

**Methods:**

The circRNA microarray was used to explore the circRNAs that were differentially expressed between ESCC and paired adjacent normal tissues. The function of circIMMP2L was validated by gain or loss of function assays. Pull‐down, RNA immunoprecipitation assays were used to demonstrate the biological mechanism of circIMMP2L. Tissue microarray (TMA), specimen, and paired plasma were investigated to evaluate the clinical significance of circIMMP2L.

**Results:**

CircIMMP2L, commonly upregulated in tumor and plasma from advanced‐stage ESCC patients and LNM patients, predicts poorer patient survival. CircIMMP2L was also found to be a significant indicator for LNM, even in the T1 stage of ESCC. CircIMMP2L depletion suppressed the malignant progression of ESCC both in vitro and in vivo. Mechanistically, cytoplasmic circIMMP2L interacted with CtBP1 and facilitated the nuclear retention of CtBP1 in a CtBP2‐independent manner. Moreover, circIMMP2L promoted the interaction of CtBP1 with HDAC1 in the nucleus, which is essential for epigenetic remodeling and transcriptional suppression of E‐cadherin and p21.

**Conclusions:**

These findings demonstrated that circIMMP2L promotes the malignant progression of ESCC mediated by CtBP1 nuclear retention and is a robust biomarker for the diagnosis, prognosis, and LNM in ESCC. Further, the findings extend our knowledge about the mechanism of circRNA regulation of gene transcription through epigenetics.

## Graphical abstract

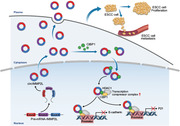

**Abbreviations**
circRNA: circular RNA
ESCC: esophageal squamous cell carcinoma
RBPs: RNA binding proteins
LNM: lymph node metastasis
ANT: adjacent normal tissues
RTCA: real‐time cell analysis
IRES: internal ribosomal entrance site
ESD: endoscopic submucosal dissection
HDACi: HDAC inhibitor
ISH: in situ hybridization

## 1 INTRODUCTION

Esophageal cancer (EC) is one of the highly aggressive tumors with a poor prognosis. According to the global cancer statistics 2020, EC is ranked the seventh most prevalent and sixth deadliest cancer.^1^ The two most common histologic subtypes of EC are esophageal squamous cell carcinoma (ESCC) and esophageal adenocarcinoma (EAC).^2^ ESCC is the most predominant EC worldwide, and the 5‐year survival ranges between 15% and 25%.^3^, ^4^ Poor outcomes in ESCC patients are associated with diagnosis at an advanced stage and lymph node metastasis (LNM). Since there is no sensitive tool for detecting ESCC at an early stage, over half of ESCC cases are diagnosed at an advanced stage. LNM is recognized as the most important independent risk factor for ESCC. Once the LNM occurs, the overall survival decreases from 70% to 18%.^5^ At present, neither the contrast‐enhanced computed tomography (CT) nor the endoscopic ultrasound (EUS) can accurately assess LNM in ESCC patients.^6^, ^7^ Endoscopic interventions are recommended for early‐stage ESCC patients (stage Tis and T1) with minimally invasive curative treatment.^8^ However, only the early stage ESCC patients with a very low rate of LNM can benefit from the endoscopic therapies. Therefore, the availability of a robust biomarker for early diagnosis and LNM can help improve the overall survival of ESCC patients and guide individualized treatment strategies.

Circular RNAs (circRNAs) are a novel class of noncoding RNAs with a covalent one‐stranded loop structure. CircRNAs are generated by directly backsplicing or skipping exons of precursor mRNA (pre‐mRNA). Numerous disordered circRNAs are expressed in different physiological and pathological processes, especially in tumorigenesis. Several circRNAs are reported to regulate cell epithelial‐mesenchymal transition (EMT),^9^ proliferation,^10^ and autophagy^11^ in various cancers. CircRNAs also interact with transcription factors to regulate their nucleoplasmic localization^12^ or directly bind to the target genes to regulate transcription.^13^ Besides, they regulate mRNA stability through posttranscriptional processing and act as “miRNA sponges” or interact with RBPs to suppress or increase the target.^14^, ^15^ CircRNAs also take part in translation progression, where they function as a brake in ribosomes to retard translation.^16^ The inherent circular characteristic renders circRNAs stable within cells and extracellular fluids such as blood, urine, and saliva.^17^ Thus, circRNAs are promising biomarkers for diseases. However, identifying essential circRNA abnormalities, their roles, and the fundamental processes underlying the progression of ESCC remains unknown.

In this study, we identified circIMMP2L as a regulator of the malignant progression of ESCC. Clinically, the unfavorable prognosis for ESCC patients is positively linked with circIMMP2L. Besides, circIMMP2L expression in tumor and plasma serves as an indicator for diagnosis and LNM of ESCC patients, even in the early stage. Functionally, circIMMP2L is upregulated and promotes invasion, migration, and proliferation in ESCC both in vitro and in vivo. Mechanistically, circIMMP2L interacts with CtBP1 in the cytoplasm to facilitate nuclear retention of CtBP1 and promote the interaction between CtBP1 and HDAC1 in the nucleus. Aberrant activity of HDAC1 deacetylates the histone H3 in the promoter regions of E‐cadherin and p21, suppressing their transcription. Our findings indicate that circIMMP2L functions as a critical molecule in ESCC malignant progression and a potential biomarker for tumor diagnosis, prognosis, and LNM.

## 2 MATERIALS AND METHODS

### 2.1 Tissues and plasma collection

The experiment was accepted by the Ethics committee of the Jiangsu Cancer Hospital, and was in obedience to relative requirements. All of the patients signed an informed consent form. Three paired Human esophageal squamous cell carcinoma (ESCC) tissues and adjacent tissues (ANT) (cohort1), 54 paired Human ESCC tissues, ANT, plasma (cohort2), and 48 T1 stage ESCC tissues (including 24 lymph node metastasis and 24 non‐lymph node metastasis) (cohort3) were taken from the the Affiliated Cancer Hospital of Nanjing Medical University between 2017 and 2020 (Nanjing, China). ESCC tissues and paired ANT were established as reliable diagnoses, with the assistance of experienced pathologists. Clinical information of the ESCC patients is summarized in Table S1.

### 2.2 Quantitative PCR and RNase R treatment

Trizol reagent has been used to isolate RNA from cells (Takara, Dalian, China). Nuclear and cytoplase extractions were used to extract nuclear and cytoplasmic RNA and protein with the cell fragmentation isolation kit (Fisher Scientific, Vilnius, Lithuania). After 1 μg of total RNA incubation at 37°C, the RNase R solution was added, the mixture was boiled for 30 min, and the reaction was allowed to proceed (Geneseed, Shanghai, China). Random hexamers were used for Reverse transcription (Takara, Liaoning, China) or oligo(dT)_18_ primers (Qiagen), and quantitative PCR was with SYBR Green master mix (Takara, Liaoning, China). Back‐splicing primers were used to measure circIMMP2L transcripts. We planned convergent primers for amplifying the exons of which the sequence was not present in the 2, 3, and 4 of the IMMP2 exons. Step one Plus Real‐Time PCR method (ABI, Foster City, CA) and its respective tools were used to do the amplification and the analysis. The expression was measured using 2^‐ΔΔCT^. Absolute quantification of circIMMP2L was performed by qRT‐PCR with respect to its standard curves. The product of circIMMP2L amplification was purified by PCR purification Kit (Qiagen). The 1 ng/μl DNA fragment stock solution was serially diluted from 1 × 10^–1^ to 1 × 10^–8^ ng/μl for a standard curve. The diluted solution was amplified using qRT‐PCR system. Use the relevant standard curve to transform the threshold period (CT) value into concentration. Primers sequences are listed in Table S2.

### 2.3 Actinomycin D assay

TE‐1 cells were placed in five wells in 24‐well plates (5 × 10^4^ cells per well). Actinomycin D was applied to cells after 24 h (2 μg/ml, Abcam, Cambridge, UK) for 0, 4, 8, 12, and 24 h, respectively. After that, the relative RNA levels of circIMMP2L and mIMMP2L were analyzed by qRT‐PCR and normalized to the values of the 0 h group.

### 2.4 RNA immunoprecipitation assay

The Magna RIPTM RNA‐Binding Protein Immunoprecipitation Kit was used in an RNA immunoprecipitation (RIP) assay (Millipore) according to the manufacturer's instructions. In brief, 2 × 10^7^ cells were incubated in lysis buffer on ice for 10 min. Magnetic beads were incubated with 5 μg antibody at room temperature. The tissue lysates were incubated with the bead overnight at 4°C. Upon the proteinase K treatment, the immunoprecipitated RNAs were extracted. The abundance of circIMMP2L was detected by qPCR. CtBP1, IgG, CtBP2, and Ago2 antibodies were used for RIP. qRT‐PCR was used to monitor the co‐precipitated RNA. The information on antibodies is listed in Table S3. The primers for qRT‐PCR are listed in Table S2.

### 2.5 Statistics

SPSS 25.0 software was used to perform all statistical analyses. Chi‐square test or Fisher's exact test were used to assess qualitative variables. Student's *t*‐test was performed when the data followed the normal distribution. Nonparametric test was used to examine the abnormal distribution variables. The method of variance (ANOVA) was performed for comparing the differences between groups. Correlation analysis was performed using the Pearson correlation coefficient method. The ROC curve was calculated for diagnosing sensitivity and specificity. The findings are indicated as the means ± standard deviation (SD). All statistical tests were two‐sided, and the *P* value < 0.05 was deemed statistically significant.

## 3 Results

### 3.1 Identification of circRNAs by circRNA microarray in human ESCC samples

**FIGURE 1:**
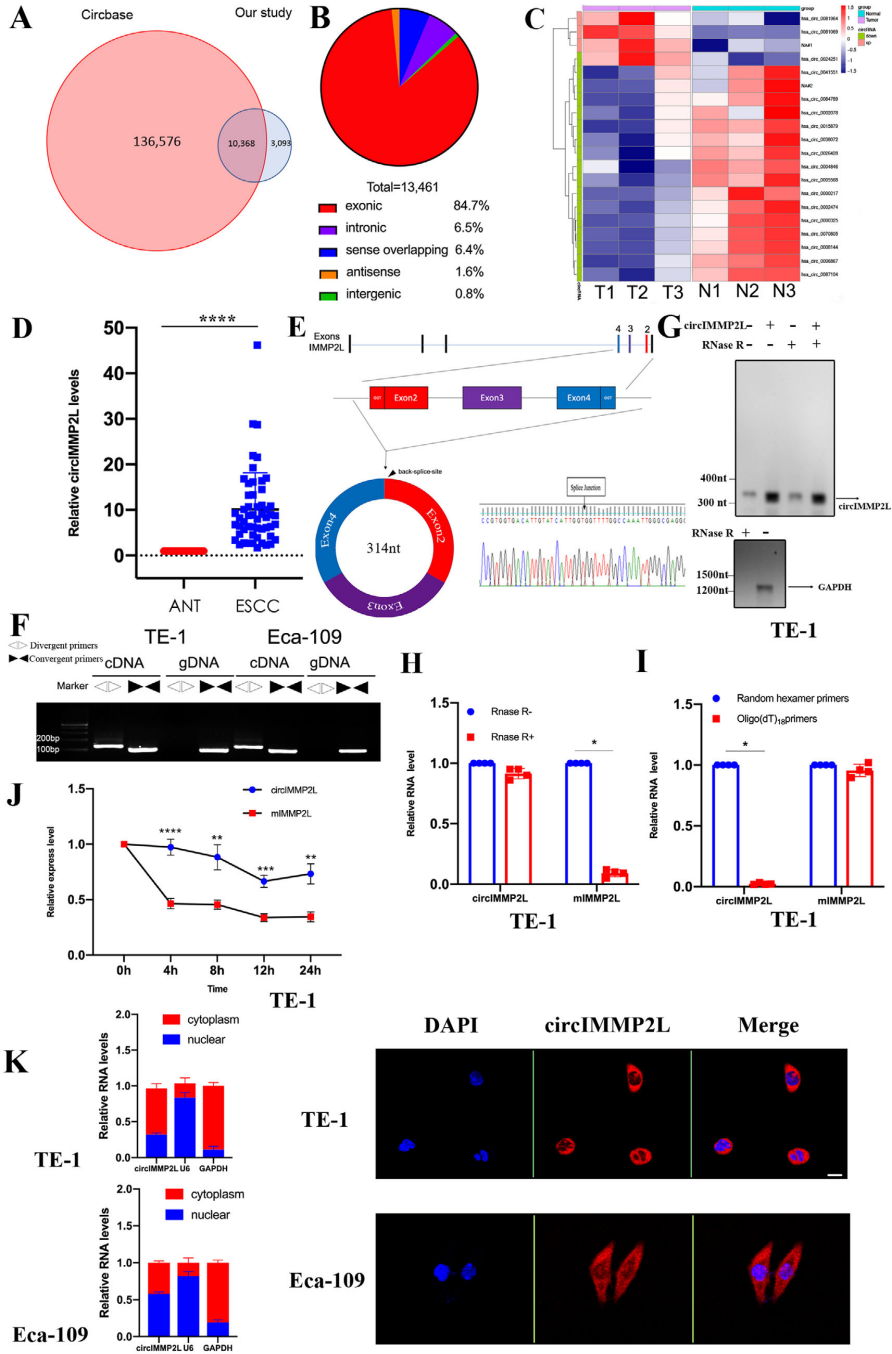
circIMMP2L is upregulated in ESCC. A, Most of the circRNAs identified overlap with circBase. B, Genomic origin of the circRNAs identified in human esophageal tissues. C, Cluster heat map of the top‐20 differentially expressed circRNAs in three paired ESSC (T1‐T3) and ANT (N1‐N3). NA, non‐annotation in circBase. D, The expression levels of circIMMP2L in 54 paired ESSC and ANT using qRT‐PCR (****p<0.0001 vs. ANT, Wilcoxon matched‐pairs signed‐rank test, n = 108). (E) Scheme illustrating the production of circIMMP2L. CircIMMP2L is formed by back splicing from exon 2 to 4 of the IMMP2L gene. The expression of circIMMP2L is validated by sanger sequencing, the arrow indicates the junction site of circIMMP2L. F, qRT‐PCR assay indicating the detection of circIMMP2L using divergent and convergent primers from cDNA or genomic DNA (gDNA) of cancer cell lines TE‐1. cDNA, complementary DNA. gDNA, genomic DNA. G, Northern blot analysis using a junction‐specific probe indicating the endogenous existence. H, Random hexamer or oligo(dT)_18_ primer used in reverse transcription experiments, and the analysis of the RNA levels by qRT‐PCR (*P < 0.05 vs. circIMMP2L, two‐tailed unpaired Student's *t*‐test, n = 4). I, PCR analysis for the expression of circIMMP2L and mIMMP2Lafter treatment with RNase R in the TE‐1 cells (*P < 0.05 vs. circIMMP2L, two‐tailed unpaired Student's *t*‐test, n = 4). J, The relative RNA levels of circIMMP2L and mIMMP2L were analyzed by qRT‐PCR after treatment with Actinomycin D at the indicated time points in TE‐1 cells (****P < 0.0001, ***P < 0.001, **P < 0.01 vs. mIMMP2L, two‐tailed unpaired Student's *t*‐test, n = 4). (K) Left, the expression levels of circIMMP2L in the nuclear and cytoplasmic fraction of TE‐1 and Eca‐109 cells. Right, FISH for circIMMP2L in TE‐1 and Eca‐109 cells. DAPI stained nuclei. Scale bar, 50μm (n = 3)

CircRNA alteration was investigated using circRNA microarray of ribosomal RNA‐depleted, and RNase R treated RNA from three paired human ESCC (T1 stage with lymph node metastasis) tissues and adjacent normal tissues (ANT) (cohort 1). A total of 13,461 circRNAs were detected (Table S4). Among them, 10,368 circRNAs are annotated in circBase (Figure 1A). The vast majority of circRNAs were generated from known gene locus excluding intergenic part, and 84.7% of the circRNAs were derived from exons and 6.5% from introns (Figure 1B). A threshold value of ≥2 (or ≤0.5)‐fold change and an adjusted *p*‐value <0.05 were used. There were nine upregulated and 63 downregulated circRNAs in the ESCC tissues compared to ANT (Figure S1A and Table S5). The most downregulated circRNAs were located in chromosome 2, reported as frequent homozygous deletion region^18^, ^19^ (Figure S2A). Among the most significant dysregulated circRNAs, nine upregulated and 11 downregulated circRNAs were further selected and used for validation (Figure 1C). Next, we examined the top 20 disordered circRNAs in 54 paired ESCC and ANT (cohort2) by quantitative reverse transcription PCR (qRT‐PCR). There were four circRNAs had differential expression (Figure S3A). CircIMMP2L was the most significantly differentially expressed circRNA in ESCC tissues compared to ANT and was selected as the candidate for further study (Figure 1D).

### 3.2 Expression and circRNA characterization of circIMMP2L in ESCC

CircIMMP2L (circBase ID: hsa_circ_0081964) was derived from exon 2 to 4 of the Inner Mitochondrial Membrane Peptidase Subunit 2 (IMMP2L) gene with a sequence of 314nt. The divergent primers were used to amplify the back‐spliced junction of circIMMP2L and validated by Sanger sequencing (Figure 1E). The full length of circIMMP2L was also confirmed by Sanger sequencing and was consistent with circBase database annotation (Table S6). The circIMMP2L nucleotide sequence is strongly conserved, with 89% homology between humans and mice (Figure S2B). Moreover, like most circRNAs,^20^ circIMMP2L was independently expressed from the linear RNA of IMMP2L (mIMMP2L) (Figure S2C). The expression of endogenous circIMMP2L was measured in ESCC cell lines and HEEC, which is the human normal esophageal epithelial cell. The results revealed that circIMMP2L was upregulated in ESCC cell lines. CircIMMP2L was highly enriched in TE‐1 cells and weakly enriched in Eca‐109 cells (Figure S2D). PCR analysis of the reverse‐transcribed RNA (cDNA) and genomic DNA (gDNA) revealed that divergent primers could amplify cDNA but not gDNA products (Figure 1F). The endogenous expression of circIMMP2L was detected in TE‐1 cells using junction‐specific probes in Northern blot assay and circIMMP2L resistant to efficient RNase R digestion (Figure 1G, H). Compared with random hexamer primers, the relative expression of circIMMP2L derived from oligo (dT)_18_ primers in reverse transcription experiments in TE‐1 cells were significantly downregulated, while mIMMP2L was not (Figure 1I). Analysis of the stability of circIMMP2L and mIMMP2L treated with Actinomycin D in TE‐1 cells, revealed that the half‐life of circIMMP2L was more stable than that of mIMMP2L (Figure 1J). Further, nuclear and cytoplasmic extraction and fluorescence in situ hybridization (FISH) demonstrated that circIMMP2L was localized in both the nucleus and cytoplasm (Figure 1K).

Collectively, circIMMP2L was upregulated, abundant, and stably expressed in ESCC.

### 3.3 CircIMMP2L is a potential biomarker for diagnosis, LNM, and prognosis in ESCC

**FIGURE 2:**
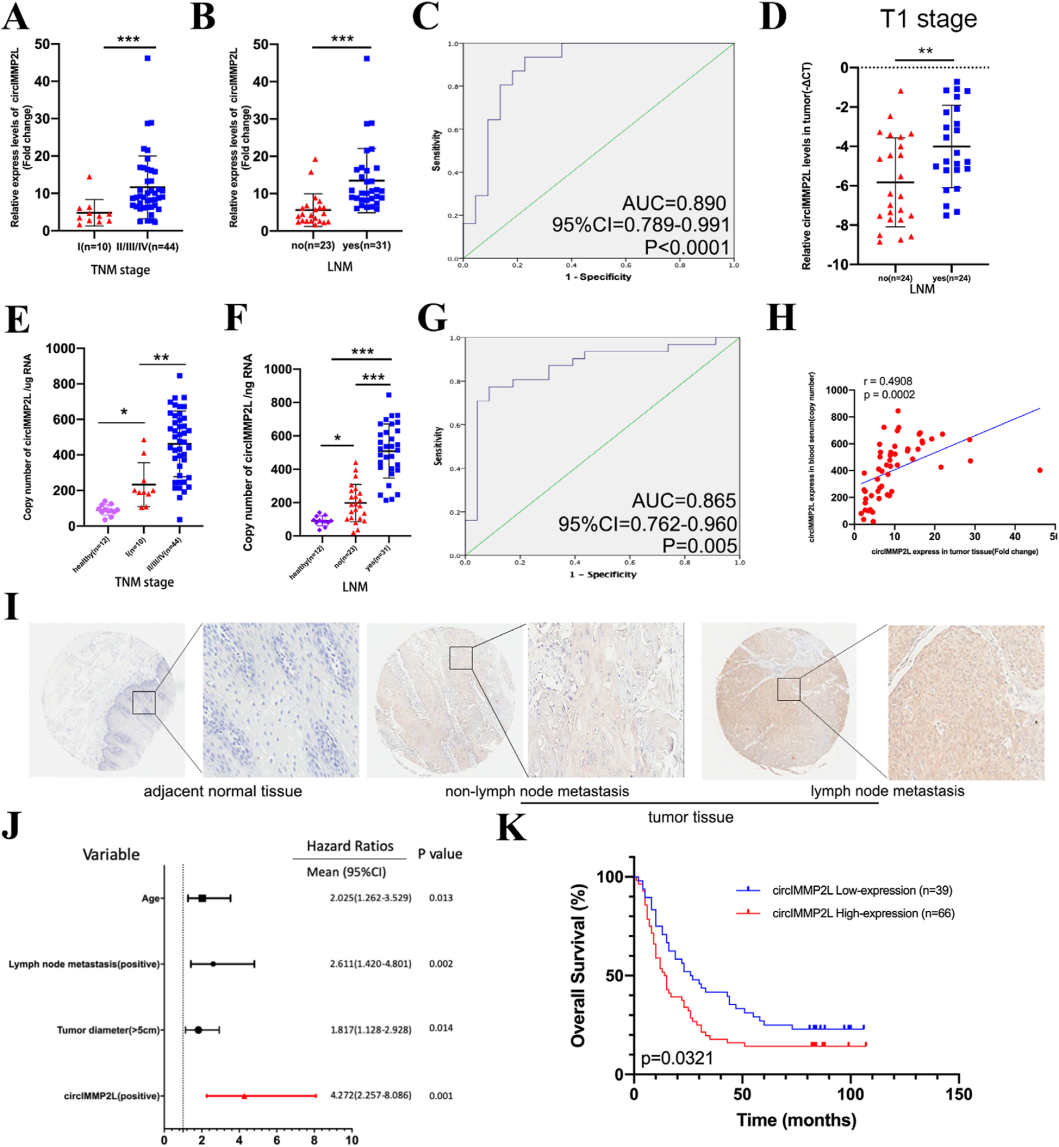
CircIMMP2L is a potential diagnosis and prognosis biomarker for ESCC in tissues and plasma. A, Scatter plots illustrating the qRT‐PCR analysis of the expression fold change for circIMMP2L in 54 ESSC and paired ANT between TNM stage I and stage II/III/IV. (***P < 0.001 vs. stage I, two‐tailed unpaired Student's *t*‐test, n = 54). B, Scatter plots illustrating the qRT‐PCR analysis of the expression fold change for circIMMP2L in 54 ESSC and paired ANT with lymph node metastasis compared to those without metastasis. (***P < 0.001 vs. no, two‐tailed unpaired Student's *t*‐test, n = 54). C, ROC curve analysis of tissues of non‐LNM and LNM ESSC patients and estimating the circIMMP2L expression. D, Differential expression of circIMMP2L between 24 non‐metastasis and 24 metastasis TNM stage IESSC tissues (**P < 0.01 vs. no, two‐tailed unpaired Student's *t*‐test, n = 48). E, Scatter plots illustrating the absolute quantification of circIMMP2L in 54 ESSC patients’ blood plasma, and between TNM stage I and stage II/III/IV. (**P < 0.01 vs. stage I, two‐tailed unpaired Student's *t*‐test, n = 54). F, Absolute quantification of circIMMP2L in 54 non‐metastasis and metastasis ESSC patients’ blood plasma (**P < 0.01 vs. no, two‐tailed unpaired Student's *t*‐test, n = 54). G, ROC curve analysis of blood plasma in non‐LNM and LNM ESSC patients estimating circIMMP2L expression. H, circIMMP2L expression levels in tissues and blood plasma, showing a significant positive correlation between paired tissues and blood plasma in 54 ESSC patients (The *P‐*value is determined by Pearson correlation analysis). I, Representative images showing the expression of circIMMP2L among adjacent normal tissue, non‐metastasis, and metastasis tissues. J, Multivariate analyses using hazard ratios for overall survival. K, Kaplan‐Meier curves showing the correlations between circIMMP2L expression and overall survival, using the Log‐rank test

Then, we explored the clinical value of circIMMP2L in the progression of ESCC. The circIMMP2L expression data of cohort2 showed that circIMMP2L was not only highly expressed in ESCC tissues, but patients with advanced TNM stage also had higher circIMMP2L expression compared with patients in early‐stage (Tis and T1N0M0; Figure 2A and Figure S4B). Since LNM is the leading cause of ESCC mortality,^21^, ^22^ we found a significant increase in circIMMP2L expression in tumor samples with LNM compared with those without LNM (Figure 2B). The ROC curve was used to assess the diagnostic sensitivity and specificity, and the Youden Index was used to pick the optimum cutoff to evaluate if the expression levels of circIMMP2L in tissues had an ESCC metastasis diagnostic benefit. The region under the curve (AUC) for circIMMP2L, which was used to differentiate ESCC from non‐LNM, was 0.890 (95%CI, 0.789‐0.991; Figure 2C).

Lymph node status is a critical factor considered for early‐stage ESCC treatment with different therapeutic modalities. Endoscopic resection is the “preferred” strategy but only benefit for early ESCC patients with a very low LNM risk. To explore whether circIMMP2L can be an indicator for LNM in early‐stage ESCC patients, qRT‐PCR was performed on another cohort containing 24 LNM and 24 non‐LNM T1 stages ESCC tissues (cohort 3) were utilized in qRT‐PCR. As expected, circIMMP2L has significantly increased in LNM T1 stage ESCC tissues (*P*‐value < 0.01; Figure 2D), indicating that circIMMP2L can be a significant biomarker for predicting LNM in early‐stage ESCC.

CircRNAs are suitable candidates for “liquid biopsy” biomarkers due to their stability and ease of detection in extracellular fluid. To determine whether the plasma level of circIMMP2L had clinical relevance in ESCC, absolute quantification PCR was used to quantify the abundance of plasma circIMMP2L in 12 healthy volunteers and 54 ESCC patients (cohort2; Figure S4A). The expression of plasma circIMMP2L in ESCC patients was significantly upregulated compared with healthy people (*P*‐value < 0.05). The abundance of circIMMP2L in early‐stage ESCC patients dramatically decreased compared to advanced‐stage ESCC patients (Figure 2E). We found that the expression levels of circIMMP2L were significantly increased in plasma derived from ESCC patients with LNM compared to healthy controls and ESCC patients without LNM (Figure 2F). ROC curve analysis revealed that the area under the curve (AUC) for circIMMP2L was 0.865 (95%CI, 0.762‐0.960), indicating that the expression of circIMMP2L in plasma was sensitive and specific in distinguishing between LNM and non‐LNM (Figure 2G). Moreover, the expression level of circIMMP2L in tissues and plasma was positively correlated (Figure 2H).

The expression levels of circIMMP2L were detected using a Tissue microarray (TMA), composed of 105 ESCC tissues and related ANT through in situ hybridization (ISH). CircIMMP2L was significantly highly expressed in ESCC tissues, particularly in ESCC with LNM (Figure 2I, Figure S4B, and Table 1, *P *< 0.0001). The expression level of circIMMP2L was an independent risk factor for overall survival (OS), identified by univariate and multivariate analyses (Figure 2J and Table S7). Furthermore, Kaplan–Meier survival curves showed that circIMMP2L high‐expression patients with a shorter OS (Figure 2K).

**TABLE 1:** Clinical characteristics of ESCC cases according to circIMMP2L expression level in tissues microarray (TMA)

		circIMMP2L		
Variables	Cases (total *n* = 105)	Low (n)	High (n)	*P*‐value
**All cases**	105	**39**	**66**	−
**Age**				
≥60	85	28	57	0.1227
<60	20	11	9	
**Gender**				
Male	77	29	48	0.8550
Female	28	10	18	
**Tumor diameter**				
≥5cm	61	10	51	0.0022^*^
<5cm	44	29	15	
**T stage**				
T1‐2 stage	27	20	7	<0.0001^*^
T3‐4 stage	78	19	59	
**Lymph node metastasis**				
positive	66	10	56	<0.0001^*^
negative	39	29	10	
**TNM stage**				
I stage	8	6	2	0.0496^*^
II and III stage	97	33	64	
**Pathologic grading**				
G1	35	19	16	0.0177^*^
G2‐4	70	20	50	

χ^2^ test was used to test the association between two categorical variables. *Statistically significant.

In general, clinical investigation above indicated that the quantity of circIMMP2L was positively related to the poor outcome of ESCC and the abundance of circIMMP2L both in tumor and plasma could serve as a biomarker for early diagnosis and LNM prediction.

### 3.4 CircIMMP2L facilitates ESCC metastasis and growth both in vitro and in vivo

**FIGURE 3:**
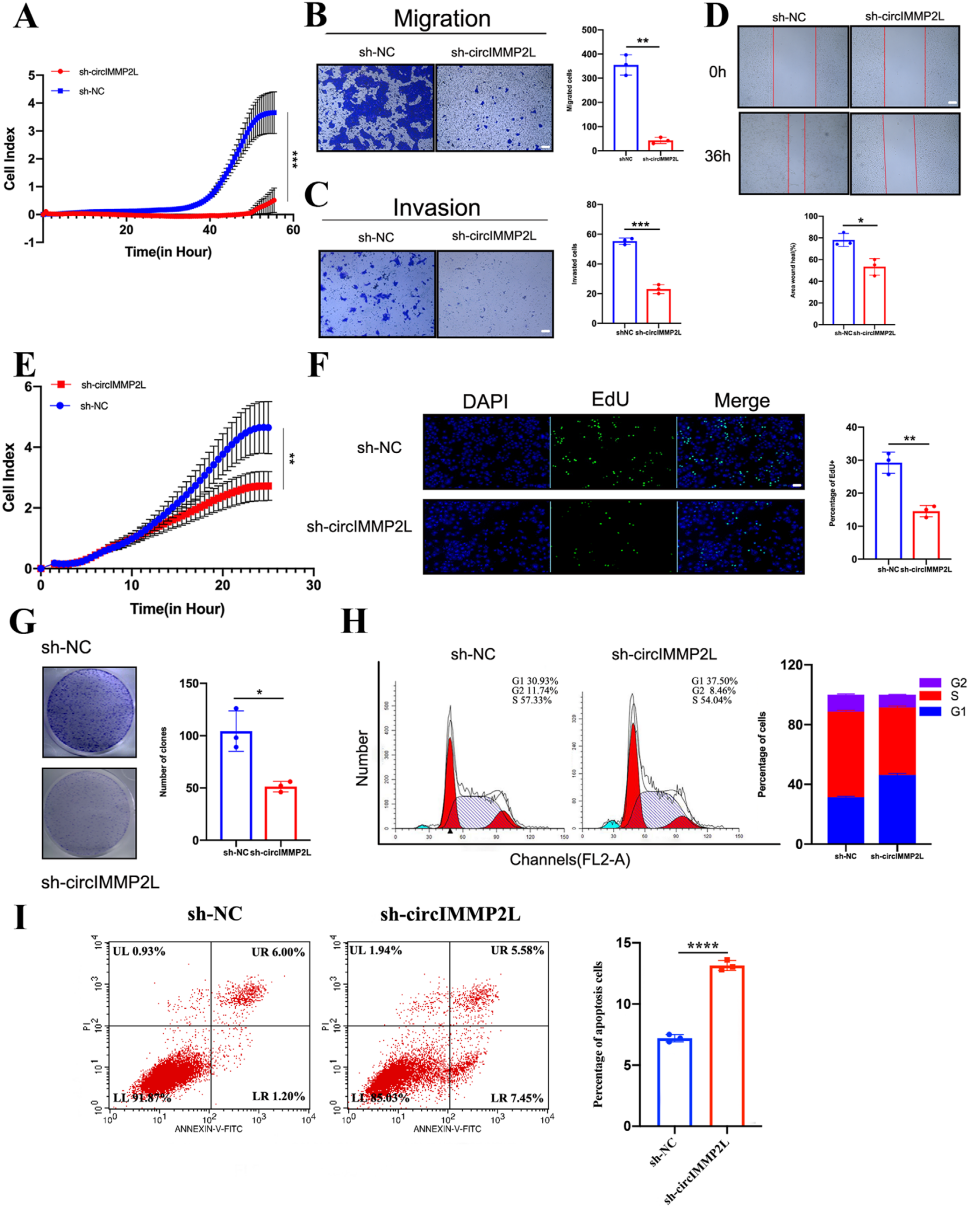
circIMMP2L promotes the metastasis and growth of ESCC cells in vitro. A, Real‐time cell analysis (RTCA) showing the invasion of TE‐1 cells stably transfected with sh‐NC or sh‐circIMMP2L and monitored by xCELLigence (***p<0.001 vs. sh‐NC, multiple comparisons two‐ANOVA, n = 3). B‐D, Transwell assay, matrigel assay and wound healing assay showing that circIMMP2L knockdown inhibits the migration and invasion ability of TE‐1 cells. Scale bar, 20μm (*P < 0.05, **P < 0.01, ***P < 0.001 vs. sh‐NC, two‐tailed unpaired Student's *t*‐test, n = 3). E, RTCA showing the growth of TE‐1 cells stably transfected with sh‐NC or sh‐circIMMP2L and monitored by xCELLigence (***P < 0.001 vs. sh‐NC, multiple comparisons two‐ANOVA, n = 3). F and G, EdU assay and colony formation assay showing that circIMMP2L knockdown inhibits the growth ability of TE‐1 cells. Left, representative images. Right, histograms of proliferation cell numbers. Scale bar, 20μm (**P < 0.01 vs. sh‐NC, two‐tailed unpaired Student's *t*‐test, n = 3). H and I, Flow cytometric analysis of cell cycle progression and cell apoptosis in TE‐1 cells stably transfected with sh‐NC or sh‐circIMMP2L. The histograms indicate the percentages of cells (***P < 0.001 vs. sh‐NC, two‐tailed unpaired Student's *t*‐test, n = 3)

To determine whether circIMMP2L was involved in the malignant phenotype of ESCC, overexpression and short hairpin RNAs (shRNAs) plasmids were established and stably transfected into cancer cells, resulting in overexpression or knockdown, respectively. Both overexpression and knockdown of circIMMP2L did not alter mIMMP2L expression (Figure S5A‐C). Real‐time cell analysis (RTCA), transwell migration assay, matrigel invasion assay, and wound healing assay revealed significant incapacitation in aggressiveness after circIMMP2L knockdown in TE‐1 cells (Figure 3A–D). Conversely, overexpressed circIMMP2L remarkably facilitated the migration and invasion of Eca‐109 cells (Figure S6A–D). RTCA, EdU assay, and colony formation assay showed dramatic growth reduction after circIMMP2L knocked down in TE‐1 cells (Figure 3E–G), while circIMMP2L overexpression in Eca‐109 cells showed enhanced cell proliferation ability (Figure S6E–G). Moreover, silencing of circIMMP2L expression decreased the percentage of TE‐1 cells in S and G2 phases and increased cell apoptosis (Figure 3H,I). However, a larger proportion of G1 phase cells and a lower percentage of apoptosis cells in circIMMP2L‐overexpression Eca‐109 cells, compared with control cells (Figure S6H,I).

**FIGURE 4:**
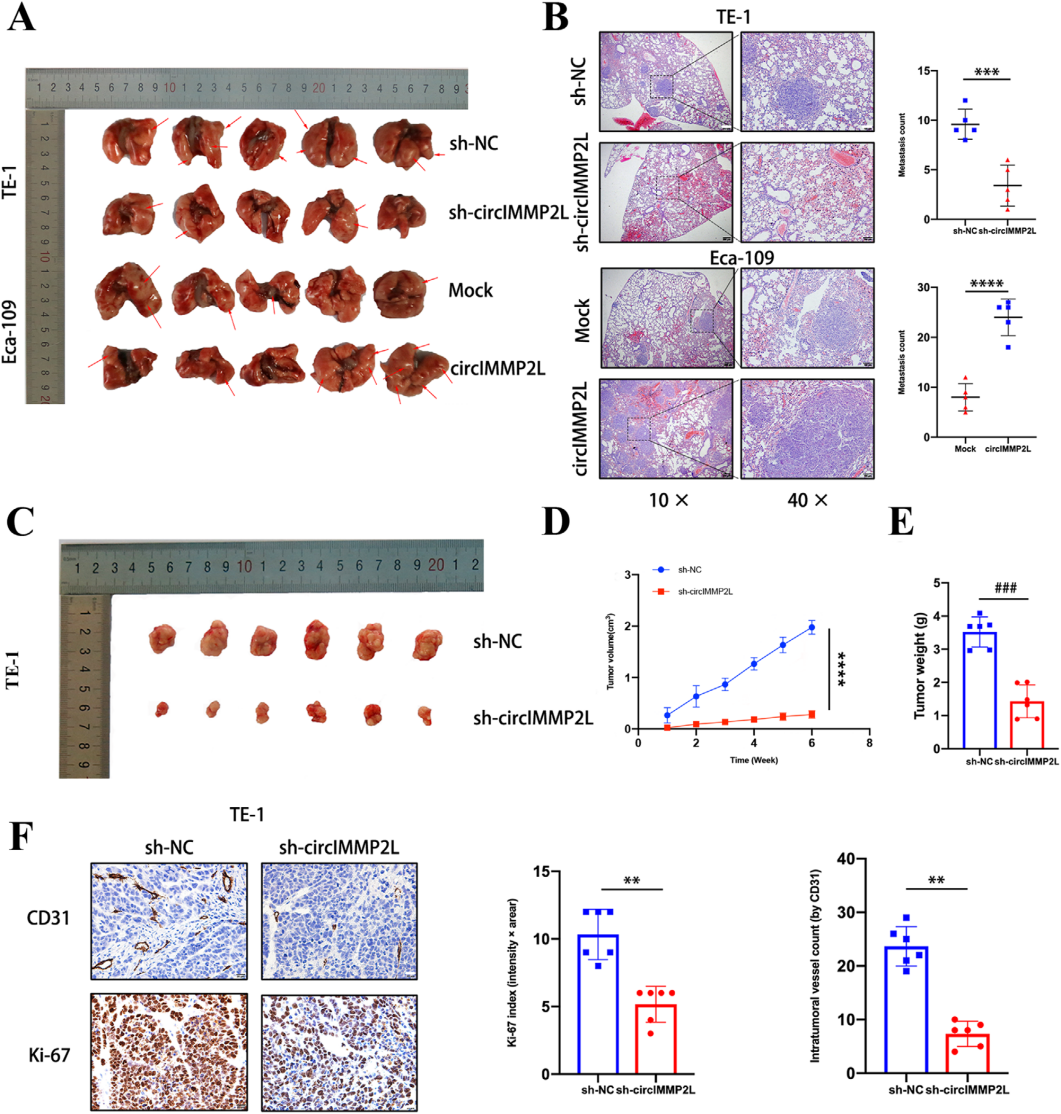
circIMMP2L promotes the metastasis and growth of esophageal squamous cancer cells in vivo. A, Representative images showing the decreased or increased tumor metastasis formed in the lungs of nude mice following vein tail injection of circIMMP2L‐knockdown TE‐1 cells or circIMMP2L‐overexpressing Eca‐109 cells. Metastases are indicated by arrows. B, Left, representative HE staining of lung metastatic lesions. 10×, scale bar, 200μm. 40×, scale bar, 100 μm. Right, quantification of metastatic nodules formed in the lungs of nude mice (***P < 0.001, ****P < 0.0001 vs. sh‐NC, two‐tailed unpaired Student's *t*‐test, n = 6). C, The representative image showing the subcutaneous xenograft tumor in nude mice derived from subcutaneous injection of control (sh‐NC) or circIMMP2L‐knockdown TE‐1 cells. D and E, The volume and weight of subcutaneous xenograft tumors of TE‐1 isolated from nude mice (**P < 0.001 vs. sh‐NC, multiple comparisons two‐way ANOVA, n = 6. ^###^ *P *< 0.001 vs. sh‐NC, two‐tailed unpaired Student's *t*‐test, n = 6). F, Representative images of CD31 and Ki67 immunohistochemical staining within subcutaneous xenografts (**p<0.01 vs. sh‐NC, two‐tailed unpaired Student's *t*‐test, n = 6)

To explore the effects of circIMMP2L in vivo, BALB/c nude mice were treated via tail vein injection of circIMMP2L knockdown TE‐1 cells, circIMMP2L overexpressed Eca‐109 cells, and paired control cells, respectively. The findings released that circIMMP2L increased the metastasis of ESCC cells in the lungs and liver (Figure 4A,B and Figure S7A‐B). A BALB/c nude mice xenograft model was established by implanting TE‐1 cells with sh‐circIMMP2L and negative control (sh‐NC), and the deletion of circIMMP2L drastically decreased tumor growth (Figure 4C–E). Immunohistochemistry performed on subcutaneous tumor tissues showed that circIMMP2L knockdown decreased the CD31‐positive intratumoral micro‐vessels and proliferation index Ki‐67 (Figure 4F).

Taken together, these findings suggested that circIMMP2L played an oncogenic role in ESCC both in vitro and in vivo.

### 3.5 CircIMMP2L interacts with and facilitates the nuclear import of CtBP1

The mechanism by which circIMMP2L regulates the malignant progression of ESCC was investigated. To investigate if circIMMP2L acts as an efficient miRNA sponge, a reciprocal immunoprecipitation experiment was performed with anti‐Argonaut 2 (Ago2) antibody, and we found that circIMMP2L just slightly adsorbed Ago2 protein compared to CDR1as, which is identified harbor over 70 miR‐7 binding sites^23^ (Figure S8A). There was no single miRNA containing more than two binding sites within circIMMP2L as predicted by StarBase,^24^ circBank,^25^ and circinteractome.^26^ Thus, we hypothesized that the biological function of circIMMP2L was not through miRNA sponging. Numerous studies have demonstrated that circRNAs have a coding potential.^27^, ^28^ Further analysis was performed by circRNADb,^29^ two internal ribosome entry sites (IRESs) were observed in the circIMMP2L sequence, suggesting the potential combining capacity of circIMMP2L with ribosomes. However, no open reading frame (ORF) was found in circIMMP2L, meaning that circIMMP2L did not present an encoding ability. Besides, a low coding_prob was using in circBank,^25^ meaning that circIMMP2L has the limited potential of being translated into a peptide (Figure S8B).

**FIGURE 5:**
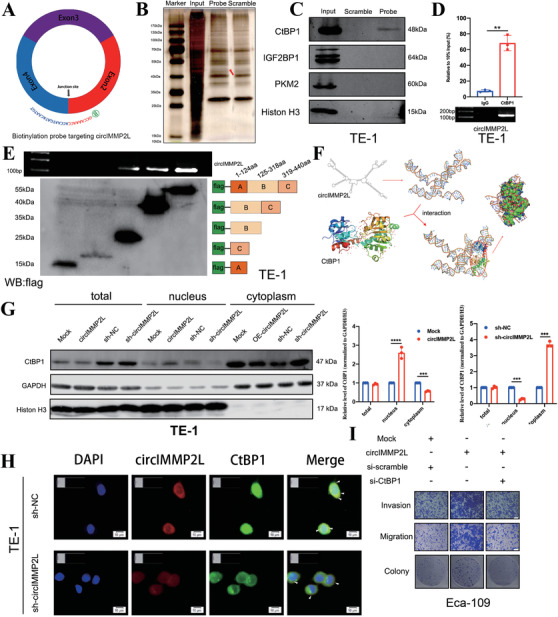
circIMMP2L interacts with and facilitates nuclear import of CtBP1 in ESCC. A, The biotin‐labeled circIMMP2L probe targets the junction site of circIMMP2L. B, Identification of the circIMMP2L‐protein complex pulled down by biotin‐labeled probe with protein extracts from TE‐1 cells. The arrow shows the additional band presented in the circIMMP2L‐protein complex. C, Western blot analysis of CtBP1, IGF2BP1, and PKM2 after pull‐down assay showing CtBP1 specifically interacting with circIMMP2L. H3 serves as a control. D, RIP assay shows the association between CtBP1 and circIMMP2L (**P < 0.01 vs. IgG, two‐tailed unpaired Student's *t*‐test, n = 3). E, In vitro binding assay depicting the recovered circIMMP2L levels from TE‐1 cells, detected by qRT‐PCR after incubation with full‐length or truncated Flag‐tagged recombinant CtBP1 protein, and validated by western blot analysis. F, Graphical representation of the three‐dimensional structures of circIMMP2L and CtBP1 docking models with a zoom‐in image of the binding interface performed using NPDock. G, Western blot analysis showing the expression of CtBP1, GAPDH, or histone H3 in total lysates or subcellular fractions of Eca‐109 cells stably transfected with empty vector (mock), circIMMP2L and TE‐1 cells stably transfected with sh‐NC, sh‐circIMMP2L (****P < 0.0001, ***P < 0.001 vs. Mock, sh‐NC, multiple comparisons two‐way ANOVA, n = 3). H, RNA‐FISH and immunofluorescence staining assay showing co‐localization of circIMMP2L (red) and CtBP1 (green) in TE‐1 cells stably transfected with sh‐NC or sh‐circIMMP2L, and DAPI stained nuclei (blue). Scale bar: 50 μm. I, Transwell assays, matrigel assays, and colony formation showing that the overexpression of circIMMP2L cloud promotes the metastasis and proliferation ability of Eca‐109 cells and which can be blocked by CtBP1 knockdown

Next, we attempted to identify the proteins that interacted with circIMMP2L. A biotin tagged probe was designed to target the back‐splicing region of circIMMP2L (Figure 5A). RNA pull‐down assay and mass spectrometry (MS) analysis were used to screen for circIMMP2L‐binding proteins (Figure 5B and Table S8). The candidates were selected based on the LFQ intensity of MS (the top 5 were selected) and their association with tumorigenesis. C‐Terminal Binding Protein 1 (CtBP1), Insulin‐Like Growth Factor 2 MRNA Binding Protein 1 (IGF2BP1), and Pyruvate Kinase M1/2 (PKM2) were identified as presumptive circIMMP2L‐binding proteins. Subsequent validation by RNA pull‐down and western blot assays in TE‐1 cells showed physical interaction of circIMMP2L with CtBP1, but not with IGF2BP1 and PKM2 (Figure 5C). Moreover, the RIP assay in TE‐1 cells indicated the enrichment of circIMMP2L in complexes precipitated with the CtBP1 antibody (Figure 5D). Subsequently, we separated the CtBP1 protein into domain A (1‐124aa), domain B (125‐318aa), and domain C (319‐440aa) based on its functional domains. In TE‐1 cells, the RIP assay indicated that domain B of CtBP1 protein was crucial for its interaction with circIMMP2L (Figure 5E). Mfold^30^ and RNA Composer^31^ were used to identify the 2D and 3D structures of circIMMP2L, respectively. The 3D structure of protein CtBP1 was derived from the Protein Data Bank (PDB) entry 4FXV. NPDock^32^ was used to calculate the in silico molecular docking between circIMMP2L and CtBP1, which supported that circIMMP2L could perfectly dock CtBP1 (Figure 5F). To further investigate the interplay between circIMMP2L and CtBP1, both the protein and mRNA levels of CtBP1 were found not altered by the expression level of circIMMP2L (Figure 5G and Figure S8C). However, ectopic elevated expression or knockdown of circIMMP2L facilitated or attenuated the translocation of CtBP1 from the cytoplasm to the nucleus, in Eca‐109 or TE‐1 cells, respectively (Figure 5G). In TE‐1 cells, dual RNA‐FISH and immunofluorescence assay confirmed the colocalization of circIMMP2L and CtBP1, and circIMMP2L depletion results in CtBP1 localizing exclusively the cytoplasm in TE‐1 cells (Figure 5H). In addition, we found that the proportion of circIMMP2L localized in the nucleus upon circIMMP2L overexpression. When we suppressed the expression level of circIMMP2L, more circIMMP2L was distributed in the cytoplasm (Figure S8D‐E).

CtBP2 is a homologous protein to CtBP1 and share high sequence homology and structural similarities with CtBP1. However, it contains a nuclear localization signal (NLS) domain at its N‐terminus.^33^ CtBP1 protein has been confirmed to interact with CtBP2 in heterodimerization for nuclear import and retention.^34^ At present, it remains unclear whether circIMMP2L acts as the scaffold structure binding with CtBP1 and CtBP2 simultaneously, and if it facilitates the interaction between the two proteins. Unexpectedly, RIP assays showed no effective binding between circIMMP2L and CtBP2 (Figure S8F). Besides, there was no significant interaction change following overexpression or knockdown of circIMMP2L (Figure S8G). Thus, circIMMP2L might promote CtBP1 nuclear accumulation in a CtBP2‐independent way.

Further, CtBP1 overexpressed plasmid and short interfering RNAs (siRNA) were used to explore if the biological function of circIMMP2L in the destructive development of ESCC relies on the CtBP1 pathway (Figure S8H‐I). Transwell, matrigel, colony assay, and EdU assays were performed in vitro (Figure 5I and Figure S9A). Moreover, the in vivo functional rescue experiments (including the BALB/c nude mice tail vein injection model and xenograft model) were also performed (Figure S10A‐F). The results of all the rescue experiments indicated that CtBP1 knockdown abrogated the effects of circIMMP2L on promoting aggressiveness and proliferation of Eca‐109 cells. Thus, we supposed that the biological function of circIMMP2L was dependent on CtBP1.

Taken together, these results indicated that circIMMP2L, at least partly, regulated the aggressiveness and proliferation of ESCC cells by interacting with CtBP1.

### 3.6 CircIMMP2L epigenetically silences the transcription of E‐cadherin and p21 by facilitating the interaction between CtBP1 and HDAC1

**FIGURE 6:**
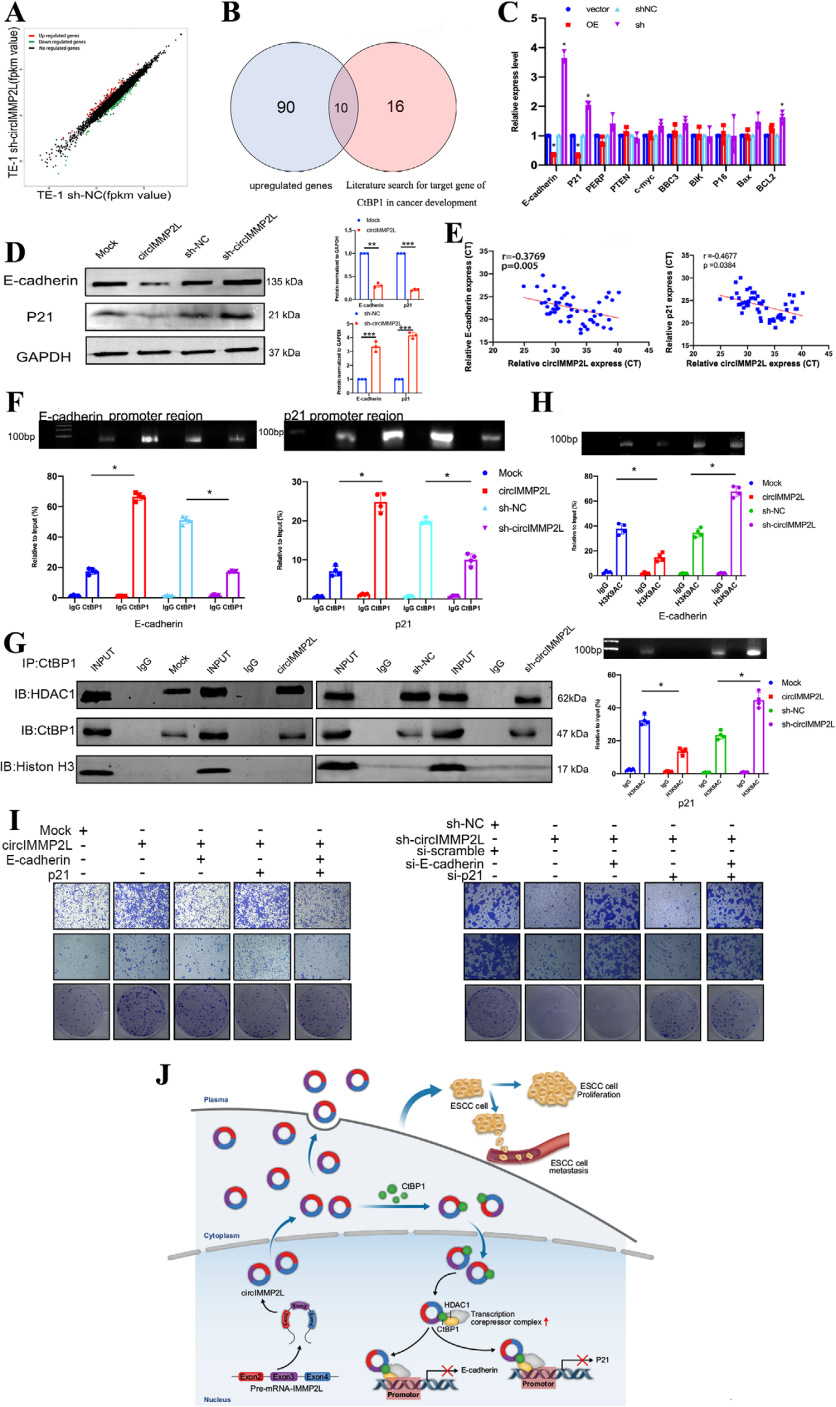
CircIMMP2L epigenetically silences the transcription of E‐cadherin and p21 by facilitating the interaction between CtBP1 and HDAC1. A, Scatter plots showing the differentially expressed genes (fold change > 2, *P *< 0.05) in TE‐1 cells stably transfected with sh‐NC or sh‐circIMMP2L. B, Venn diagram showing upregulated genes from the mRNA‐microarray assay, and based on candidate cancer‐associated CtBP1‐downstream genes. C, qRT‐PCR assays showing the expression of ten genes in TE‐1 cells stably transfected with sh‐NC or sh‐circIMMP2L and Eca‐109 cells stably transfected with the vector (mock) or circIMMP2L (*P < 0.05 vs. Mock or sh‐NC, two‐tailed unpaired Student's *t*‐test, n = 4). D, Western blot assays showing the expression of E‐cadherin and P21 in TE‐1 cells stably transfected with sh‐NC or sh‐circIMMP2L and Eca‐109 cells stably transfected with the vector (mock) or circIMMP2L. E, E‐cadherin and P21 expression showing a significant negative correlation with circIMMP2L expression in 54 ESSC tissues. (The *P‐*value was determined by Pearson correlation analysis). F, ChIP‐qRT‐PCR of CtBP1 of the promoter regions of E‐cadherin or P21 gene locus in TE‐1 cells stably transfected with sh‐NC or sh‐circIMMP2L and Eca‐109 cells stably transfected with the vector (mock) or circIMMP2L (*P < 0.0.05 vs. Mock or sh‐NC, two‐tailed unpaired Student's *t*‐test, n = 4). G, Co‐IP and western blot assays showing differential interaction levels between CtBP1 and HDAC1 in Eca‐109 cells stably transfected with the vector (mock) or circIMMP2L, and TE‐1 cells stably transfected with sh‐NC or sh‐circIMMP2L. H, ChIP‐qRT‐PCR of H3K9AC in the promoter regions of E‐cadherin or P21 gene locus in TE‐1 cells stably transfected with sh‐NC or sh‐circIMMP2L and Eca‐109 cells stably transfected with the vector (mock) or circIMMP2L (*P < 0.0.05 vs. Mock or sh‐NC, two‐tailed unpaired Student's *t*‐test, n = 4). I, Transwell assays, Matrigel assays, and colony formation showing that overexpression of circIMMP2L promotes the metastasis and proliferation ability of Eca‐109 cells, metastasis is blocked by over‐expression of E‐cadherin, and growth was blocked by P21‐overexpression. J, CircIMMP2L regulates the E‐cadherin and p21 transcription in ESCC through an epigenetic manner, and as a biomarker for the diagnosis, prognosis, and lymph node metastasis, even in an early stage

CtBP1 functions primarily as a transcriptional corepressor.^35^ Thus, we explored the downstream targets of circIMMP2L at the transcriptional level by mRNA microarray in TE‐1 cells (Figure 6A). circIMMP2L knockdown revealed that there were 90 upregulated genes (fold change > 2, *P* < 0.05; Table S9). A comprehensive analysis of the 90 genes and the target genes of CtBP1,^33^ released that 10 candidate genes were identified (Figure 6B). Further qRT‐PCR and western blot validation showed that E‐cadherin and p21 were the primary downstream targets of circIMMP2L (Figure 6C‐D)

In addition, the expression of E‐cadherin and p21 were negatively related to the circIMMP2L expression levels in 54 ESCC tissues (cohort2) (Figure 6E). The percentages of E‐cadherin and p21‐positive cells upregulated within circIMMP2L‐silencing subcutaneous tumor tissues (Figure S11A). CtBP1 effectively halted the transcriptional activity at the promoter regions of target genes.^33^, ^36^ Thus, we further investigated if the aberrant expression of circIMMP2L could affect CtBP1 occupancy of the promoter regions in E‐cadherin and p21. Chromatin immunoprecipitation (ChIP) established that overexpression of circIMMP2L in Eca‐109 cells significantly increased the binding ability of CtBP1 on the promoter regions of E‐cadherin and p21. And the silencing of circIMMP2L in TE‐1 cells showed the opposite results (Figure 6F).

CtBP1 directly interacts with HDAC1 or is recruited as part of the HDAC1/CoREST/LSD1 corepressor complex to suppress the transcription of relative gene.^33^ HDAC1 is the core machinery of the corepressor complex and the repression activity of CtBP1 relies on it.^34^ HDAC1 is considered a transcriptional co‐repressor by removing acetyl groups from histone tails.^37^ HDAC1 has been reported to negatively regulate E‐cadherin^36^ and p21^38^ expression by deacetylating histone H3 at lysine 9 of the promoter regions. The expression levels of circIMMP2L or CtBP1 have not regulated the endogenous expression level of HADC1 (Figure S11B‐C). Endogenous interaction between CtBP1 and HDAC1 was reported before, and this interaction was also observed in Eca‐109 and TE‐1 cells (Mock and sh‐NC group, respectively) in our immunoprecipitation (IP) assay, and ectopic overexpression of circIMMP2L promoted their interaction (Figure 6G and Figure S11D). However, opposite results were observed following circIMMP2L knockdown. ChIP assay showed that circIMMP2L over‐expression increased HDAC1‐mediated H3K9 de‐acetylation in the promoter regions of E‐cadherin and p21 and opposite results were observed following circIMMP2L silencing (Figure 6H).

Rescue experiments in ESCC cells were performed to clarify the link among circIMMP2L, E‐cadherin, p21, and malignant progression. Overexpression plasmids and siRNAs of E‐cadherin and p21 were effectively designed (Figure S11E‐F). Transwell and matrigel assays further confirmed that the silencing of E‐cadherin abolished the effects of circIMMP2L on ESCC metastasis. Colony formation and EdU assays revealed that the silencing of p21 rescued the effects of circIMMP2L on proliferation. In contrast, overexpression of E‐cadherin and p21 rescued aggressiveness and growth inhibition (Figure 6I and Figure S12A).

These results showed that E‐cadherin and p21 transcription was suppressed via circIMMP2L‐CtBP1‐HDAC1 axis‐mediated epigenetic remodeling.

## 4 DISCUSSION

Numerous circRNAs have been characterized and identified to regulate cancer progression, but not in early diagnosis and LNM of ESCC. Our research identified circIMMP2L, which was derived from two to four exons of the IMMP2L gene. circIMMP2L was elevated in both tumor tissues and plasma of ESCC patients and significantly increased in LNM and advanced TNM stages. CircIMMP2L was sensitive and specific for the diagnosis and LNM prediction of ESCC, and even in the T1 stage. CircIMMP2L functioned as a tumor promoter and facilitated the invasion, migration, and proliferation of ESCC, both in vitro and in vivo. Upregulated circIMMP2L in the cytoplasm interacted with CtBP1 and led to nuclear import and retention of CtBP1 via a CtBP2‐independent manner. CircIMMP2L promoted the interaction between CtBP1 and HDAC1 in the nucleus, causing more HDAC1 to occupy the promoter regions of E‐cadherin and p21. The dysregulated HDAC1 de‐acetylated histone H3 in the promoter regions, and inhibited E‐cadherin and p21 transcription. The downregulated expression of E‐cadherin and p21 directly led to the malignant progression of ESCC.

The diagnosis of ESCC is often delayed because of the few symptoms presented in the early stage. Therefore, a specific and sensitive biomarker for early diagnosis of ESCC is essential to improve the screening and treatment strategies. Biomarkers that can be detected in a non‐invasion test are even more acceptable. In this study, circIMMP2L was revealed to be detectable and stable in tumor tissues and in plasma of ESCC patients. Endoscopic submucosal dissection (ESD) is considered the first treatment option for T0/T1 stage ESCC in Asia and Europe since it is associated with lower mortality and morbidity rates.^39^ However, ESD treatment in early‐stage ESCC is only acceptable for patients with a very low risk of LNM.^40^ It has been reported that the prevalence of LNM in ESCC is 25% for T1a stage tumors and 33.3% for T1b.^41^ Therefore, a sensitive biomarker like circIMMP2L can be used to predict the potential of LNM in early‐stage ESCC, and this presents a new tool for predicting early‐stage ESCC patients who are eligible for ESD.

CtBP1 is a critical transcriptional corepressor protein, leading to transcriptional imbalance. CtBP1 induced a wide range of pro‐tumorigenic and cancer stem cell relative functions through transcriptional regulation of gene networks in the nucleus.^42^ CtBP1 interacts with CtBP2 as heterodimerization for nuclear import in a classical manner.^43^ CtBP1 also binds to transcriptional repressors such as zinc finger E‐box proteins (ZEB1/2) for transportation into the nucleus.^44^ SUMOylation and ubiquitination are the other two ways for CtBP1 nuclear import.^43^, ^45^ The comprehensive analysis revealed that circIMMP2L‐dependent nuclear import of CtBP1 is essential for its nuclear retention. Therefore, the mechanism of CtBP1 transportation in the context of circIMMP2L should be clarified further.

Epigenomic alterations including changes in DNA methylation, histone acetylation, and RNA editing have been observed in ESCC and contribute to its carcinogenesis and progression.^46^, ^47^ The histone acetylation/deacetylation is a primary epigenetic transcriptional regulation system of gene expression.^48^ Abnormal expression and activity of HDACs and acetylated core histones regulate the gene transcription and chromatin remodeling.^49^ HDAC1 is frequently aberrantly expressed in ESCC with histone H3 hypoacetylation. MS‐275, an HDACs inhibitor (HDACi), has been confirmed to rescue the proliferation of ESCC cells.^50^ Increased emphasis has been placed on the ability of noncoding RNA to modulate histone acetylation and, on their role as epigenetic modifiers. LncPRESS1, a long non‐coding RNA (lncRNA) decreased the localization SIRT6 in chromatin to keep the enrichment of H3K56 and H3K9 acetylation at promoters of pluripotency.^51^ MiR‐449a could target and repress the expression of HDAC1‐3 in hepatocellular carcinoma.^52^ Re‐expression of p21 is an example of the gene de‐repression of post‐HDACi treatment.^53^ Our study clarified a novel epigenetic pattern of circRNAs involved in the epigenetic alterations of gene transcription.

## 5 CONCLUSIONS

Taken together, we demonstrate that circIMMP2L is an important oncogenic circRNA, which is negatively associated with overall survival in ESCC and acts as an early diagnosis and LNM biomarker both in tumor tissues and plasma. Upregulated circIMMP2L facilitates the invasion, migration, and proliferation of ESCC both in vivo and in vitro. CircIMMP2L may combine with CtBP1 in the cytoplasm promotes nuclear retention of CtBP1 in a CtBP2‐independent manner. circIMMP2L facilitates the interaction between CtBP1 and HDAC1 in the nucleus, and HDAC1 deacetylates the promoter histone H3 in lysine 9 of the promoter region to inhibit E‐cadherin and p21 transcription. This study reveals a novel transcriptional regulation of circRNAs, which is epigenetically modified and provides a potential and promising biomarker to enhance the survival of ESCC patients (Figure 6J).

## ETHICAL APPROVAL AND CONSENT TO PARTICIPATE

This study has been approved by the Ethics Committee of the Nanjing Medical University Affiliated Cancer Hospital and was performed in accordance with the provisions of the Ethics Committee of Nanjing Medical University.

## CONFLICT OF INTEREST

The authors have declared no conflict of interest.

## AUTHOR CONTRIBUTIONS

Feng Jiang, Gaochao Dong, and Yingkuanliang conceived and designed the experiments; Yingkuan Liang, Qixing Mao, Lin Wang, Wenjie Xia, Hui Wang, and Bing Chen performed the experiments; Rutao Li and Te Zhang analyzed the data; Yingkuan Liang and Gaochao Dong wrote the paper. All authors read and approved the final manuscript.

## Supporting information



Supporting Information

Supporting Information

Supporting Information

## Data Availability

The circRNA microarray is available in the Gene Expression Omnibus (GEO) (https://www.ncbi.nlm.nih.gov/geo/query/acc.cgi) under accession numbers GSE150476, un‐release now). The source data of other figures are provided as a Source Data file. All other data are available from the authors upon reasonable requests.
