# Supplementary material for: CircIMMP2L promotes esophageal squamous cell carcinoma malignant progression via CtBP1 nuclear retention dependent epigenetic modification
Source: Clin Transl Med. 2021 Sep 26;11(9):e519. doi: 10.1002/ctm2.519 (PMC8473481; doi:10.1002/ctm2.519)
Supplement: Supplementary file 1 — Supporting Information [file CTM2-11-e519-s001.pdf]

Supplementary Method

Cell cultures The TE-1 cells (RRID: CVCL_1759) and Eca-109 cells (RRID: CVCL_6898) were obtained from the Chinese Academy of Sciences Cell Bank and were authenticated by using DNA fingerprinting or isotyping analysis, and were tested negative for mycoplasma contamination. TE-1 and Eca-109 cells were cultured in RPMI-1640 medium (Keygen Biotech, China) supplemented with 10% fetal bovine plasma (Gibco, Grant Island, USA). In a humidified 37°C incubator, cells were stored in a 5 percent CO2 enviroment. Guangzhou Biotech Corp. has authenticated the cells within the last three years and has found no mycoplasma.

Microarray analysis. circRNA microarray analysis was performed using Arraystar Human circRNA Array V2. Total RNA from ESCC and paired ANT was quantified using the NanoDrop ND-1000. Briefly, total RNAs samples were digested with Rnase R (Epicentre, Madison, WI, USA) to exclude linear RNAs. The remaining RNAs were amplified and transcribed into fluorescent cRNA utilizing a random priming method (Arraystar Super RNA Labeling Kit; Arraystar). cRNAs were labeled and hybridized onto the Arraystar Human circRNA Array V2 (8x15K, Arraystar). Then the arrays were scanned by the Agilent Scanner G2505C. Agilent Feature Extraction software (version 11.0.1.1) was used to analyze the results. Quantile normalization and subsequent data processing was performed via the R software limma package. Normalized Intensity of each group (averaged normalized intensities of replicate samples, log2 transformed) were analyzed by paired t-test. The cutoffs were $p \leq 0.05$ and $|FC| \geq 2.0$.

Over-expression or knockdown of genes Human circIMMP2L linear sequence was obtained from esophageal squamous cell carcinoma tissues by PCR (Table S2) and inserted into plasmid vector pcDNA 3.1 (Hanbio, shanghai, China). Human E-cadherin cDNA and Flag tagged CtBP1 were amplified with PCR primers (Table S2) and subcloned into

pcDNA3.1 empty vector (Hanbio). p21 cDNA was direct biosynthesis *in vitro* and inserted into plasmid vector pcDNA 3.1 (Hanbio). The small interfering RNA (siRNA) of circIMMP2L, CtBP1, FUS, E-cadherin, and p21were provided by RiboBio (Guangzhou, China). The target sequences were supplied in Table S2. According the target sequence of si-circIMMP2L_1, cloned a short hairpin RNA (shRNA) (sh-circIMMP2L) into pGFP-u6 vector. The transient transfection of the shRNA or the overexpressing plasmids were performed using the Lipofectamine 3000 kit (Invitrogen, Carlsbad, CA) according to manufacturer's instructions, and the transient transfection of siRNA were performed using the Lipofectamine iMax kit(Invitrogen) according to manufacturer's instructions.

Actinomycin D assay TE-1 cells were planted in 5 wells in 24-well plates (5 × 104 cells per well). 24 hours later, the cells were exposed to actinomycin D (2μg/ml, Abcam, Cambridge, UK) for 0h, 4h, 8h, 12h and 24h, respectively. After that, the cells were harvested and the relative RNA levels of circIMMP2L and mIMMP2L were analyzed by qRT-PCR and normalized to the values measured in the mock treatment group (the 0h group).

Transwell and Matrigel assay For migration assay, 4×10^4 cells were seeded into the upper transwell assay chambers with 8μm pore filters (Millipore) in serum-free medium. For invasion assay, 4×10^4 cells were seeded into the upper matrigel assay chambers with a matrigel-coated membrane (Corning, Massachusetts, USA) in serum-free medium. The lower chamber contained medium with 10% FBS as chemokine. After incubation for 24 hours for migration and 48h for invasion at 37°C, non-migrating or non-invading cells were gently removed and cells migrated to the bottom of the membrane were fixed with 4% paraformaldehyde, stained with crystal violet solution for 30 min, and visualized under a microscope at × 100 magnification.

Wound-healing assay Transfected cells were cultured in 6-well plates. After the cells reached 90% confluence, a standard 200μl pipette tip was subsequently utilized to scratch linear wounds. In addition, the cell monolayers were cultivated in FBS-free medium. After scratching, the images of the wound closure were captured at 0, and 36h.

Real-time cell analysis (RTCA) For migration assay, the "xCELLigence" system (Roche Applied Sciences and ACEA Biosciences) including 16-well CIM-16 (Roche Diagnostics GmbH, Mannheim, Germany) is used. Initially, 165μl medium and 30μl serum-free medium were added to the lower and upper chambers respectively and the CIM-16 plate was locked at 37°C and 5% CO_2 30 minutes to obtain equilibrium. 4×10^4 cells resuspended in serum-free100μl medium to seed in the upper chamber. To monitor the cellular proliferation we utilized the "xCELLigence" system including 16-well E-plates for continuous and label-free detection. After setting up the program, 50 μL of the culture medium per well was dropped into the E-plate to plot the baseline, followed by seeding 8000 cells in each well. The cells were placed at room temperature for 30 min to attach to the E-plate before subsequent detection. Cells incubate at 37°C and 5% CO_2 and Cell Index (CI) was monitored every 30 min for the duration of the experiment.

EdU assay The ability of cell proliferation was tested by EdU (5-ethynyl-20-deoxyuridine) assay using Cell-Light EdU DNA Cell Proliferation Kit (Keygen Biotech). TE-1 and Eca-109 cells (1×10^4) were seeded in each well of 96-well plates for transfection with negative control (sh-NC), sh-circIMMP2L, empty vector (Mock), and circIMMP2L. After incubation at 37 °C and 5% CO2 for 48 h, cells were added with 50 mM EdU and incubated for another 2 h. Cells were then fixed with 4% paraformaldehyde and stained with Apollo Dye Solution for proliferating cells. Nucleic acids in all cells were stained with Hoechst 33342. The cell proliferation rate was calculated according to the manufacturer's instructions.

In vivo animal model and growth, metastasis assays Thirty female BALB/c nude mice weighing 18–22g were randomly assigned to six groups. Eca-109-Mock, Eca-109-circIMMP2L, TE-1-sh-NC, and TE-1-sh-circIMMP2L cells were prepared as a suspension of 4×10^5 cells in 200μl saline respectively and injected into the tail vein. Mice were sacrificed at 6 weeks post injection and examined microscopically by H&E staining for the development of lung metastatic and liver metastatic foci. Subcutaneous xenografts were established in the unilateral axillary of nude mice using Eca-109-Mock or Eca-109-sh-circIMMP2L cells. Tumor development was observed every week with a caliper. All mice were sacrificed 6 weeks later, measure the weight of subcutaneous xenograft tumors and pieces of tumor tissues were applied to establish orthotopic implanted models. The animal study was carried out according to the State Food and Drug Administration of China regulations on animal care. Animals were sorted only by treatment, there was no exclusion or inclusion of an animal was predetermined.

Western blotting Briefly, total protein of cells was extracted using RIPA (Thermo Fisher Scientific, Waltham, USA) with a cocktail of proteinase and phosphatase inhibitors (Thermo Fisher Scientific) according to its protocol. Equal quantities of protein lysates were resolved by SDS-PAGE gels and then transferred on a PVDF membrane (Millipore, Massachusetts, USA). The membranes were hybridized at room temperature with a secondary antibody for 1 hour after incubation with a parimary antibody at 4°C overnight. ECL identification was used to visualize blots (Thermo Fisher Scientific). The antibody information is listed in Table S3.

RNA pull-down The Biotin-labled RNA probes of circIMMP2L and scramble were synthesized by GenePharma Company (Suzhou, China). A Biotinylated Protein Interaction

Pull-Down Kit was used in RNA pull-down assay (Thermo Fisher Scientific). In brief, 2×107 cells incubated in lysis buffer on ice for 30min. The streptavidin-coated magnetic beads were incubated with biotinylated probes at room temperature for 30 min. The beads-probe complex was added to lysis, and mixed at 4°C for 2h. Proteins attached to the sealed beads were eluted. The proteins were analyzed by mass spectrometry (MS) analysis, silver staining, and western blot assay. The sequences of probes were provided in Table S8.

RNA-Fluorescence in situ hybridization assay and Fluorescence immunocytochemical staining RNA-Fluorescence in situ hybridization (FISH) assays were performed using an RNA-FISH kit (GenePharma, China) according to the manufacturer's instructions. Cy3-labeled antisense probe was synthesized by GenePharma company (Suzhou, China) against the junction site of circIMMP2L. The sequence was listed in Table S2. In briefly, TE-1 cells were fixed with 4% paraformaldehyde. After pre-hybridization with 1× PBS/0.5% Triton X-100, cells were blocked and hybridized in hybridization buffer with Cy3-labeled probe at 37 °C overnight. Then cells were incubated with specific antibodies for CtBP1 (1:200 dilution) at 4 °C overnight. Cells were stained with DAPI (300 nmol/L). The probes were provided in Table S2, and antibody information was listed in Table S3.

Microarray assay The Agilent human mRNA array was designed with 8 identical arrays per slide (8 × 60 K format), with each array containing probes interrogating approximately 27958 Entrez Gene RNAs and 7419 long intergenic noncoding RNA. The array also contains 1280 Agilent control probes. Total RNA containing small RNA was extracted from cells by using TRIzol reagent (Invitrogen) according to the manufacturer's protocol. The purity and concentration of RNA were determined from optical density 260/280 readings using a spectrophotometer (NanoDrop ND-1000). RNA integrity was determined by 1% formaldehyde denaturing gel electrophoresis.

Tissue microarray and in situ hybridization assay Tissue microarray (TMA) (Cat: HEsoS180Su05) was obtained from Outdo Biotech Co. Ltd. (Shanghai, China). 105 paired of esophageal squamous cell carcinoma (ESCC) tissues and their paired adjacent normal tissues (ANT) were used to construct the TMA. Special biotin labelled probe against circIMMP2L was synthesized by GenePharma company. The sequence was provided in Table S2. After incubating with the biotin labelled probe, the TMA was then stained using UltraSensitive^{TM} SP kit (Cat: KIT-9709/9719, Maxim, Fuzhou, China). Yellow, yellowish-brown, or darker immunohistochemical staining was considered as positive. The TMA staining scores were evaluated by two independent observers blinded to the clinicopathological data. The staining scores were based on two indicators: the proportion of positively stained cells and the staining intensity. The proportion of positively stained cells was evaluated with five scoring levels: 0, $<10\%$; 1, 10–25%; 2, 25–50%; 3, 50–75%; and 4, $>75\%$. The staining intensity was scored with the following point system: 0 (no staining), 1 (yellow), 2 (yellow-brown) and 3 (dark brown). The products of the above two indicators were considered the total score.

Supplementary Figure Legend

A

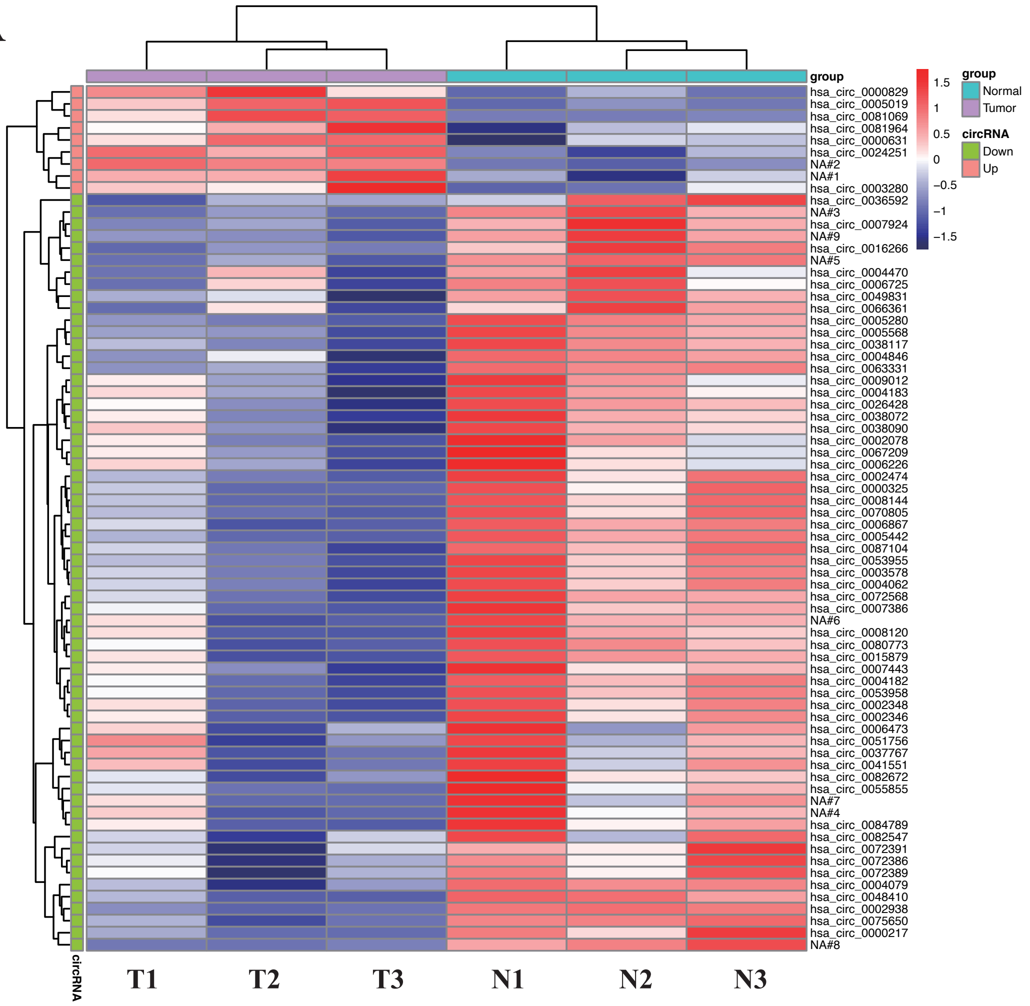

Figure S1 (A) Cluster heat map of the 72 differentially expressed circRNAs in three paired ESSC (T1-T3) and ANT (N1-N3), NA, no annotation in circBase database.

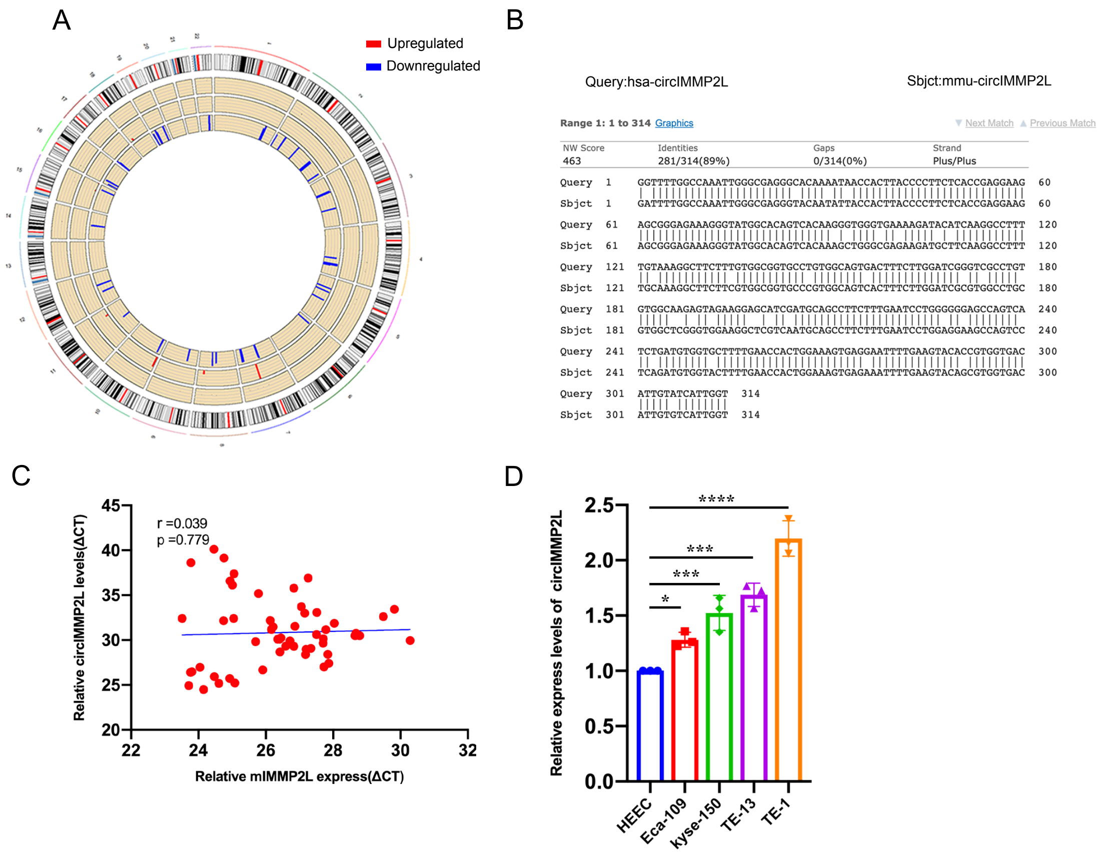

Figure S2 (A) Circos plot shows the differentially expressed circRNAs in three paired ESSC (T1-T3) and esophageal adjacent tissue(N1-N3). (B) CircIMMP2L homology analysis of human and mouse genome was conducted by using the Basic Local Alignment Search Tool (BLAST). (C) circIMMP2L expression showed non-significantly correlated with mIMMP2L expression in 54 paired ESSC and ANT (The *p* value was determined by Pearson correlation analysis). (D) The expression of circIMMP2L in HEEC and kinds of ESSC cell lines (*****p*<0.0001, ****p*<0.001, **p*<0.05 vs. HEEC, multiple comparisons two-ANOVA, n=3).

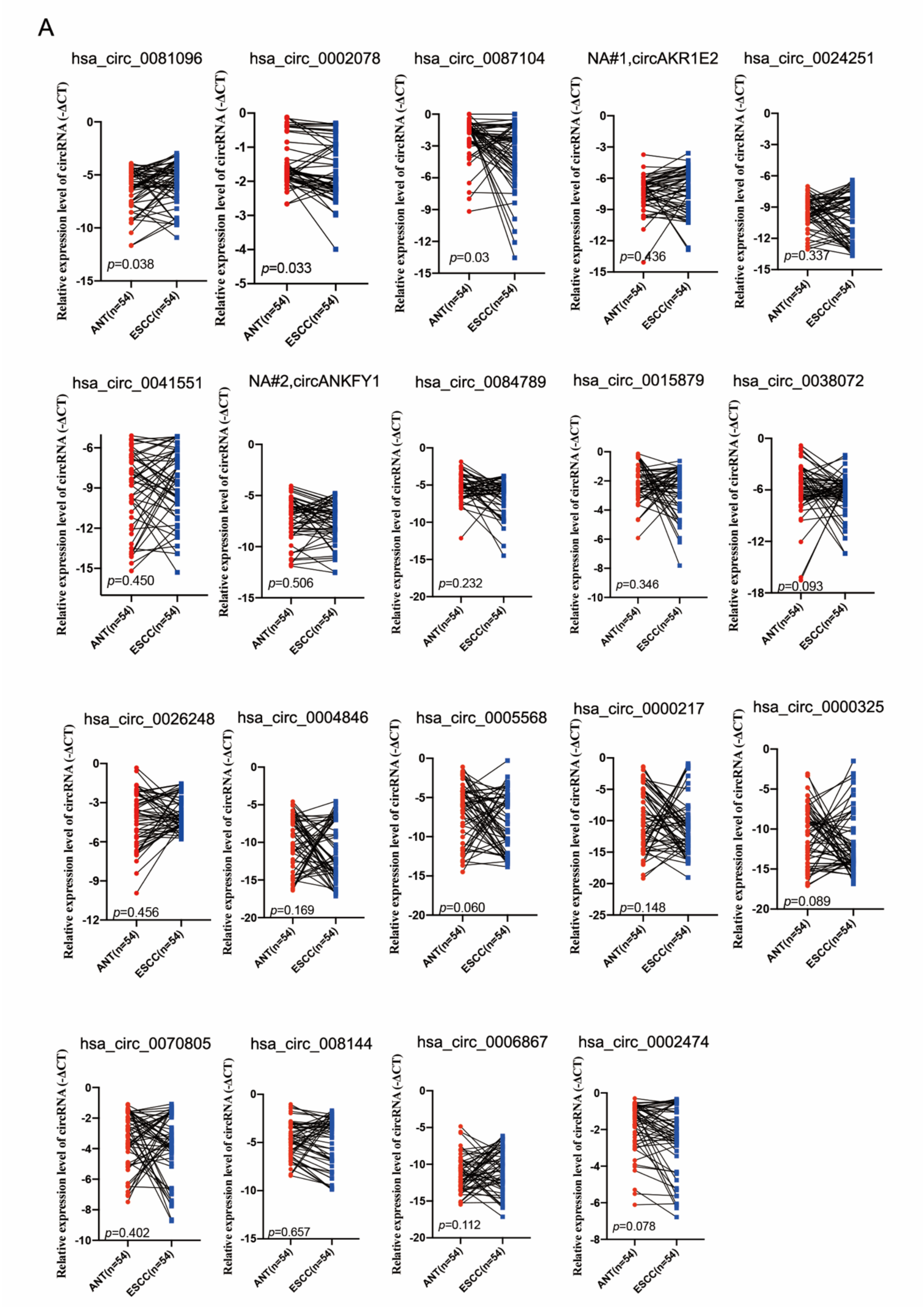

Figure S3 (A) The expression levels of 19 candidates in 54 paired ESSC and ANT using qRT-PCR (normalized to GAPDH mRNA, Wilcoxon matched-pairs signed-rank test, n=108；NA, no annotation in circBase).

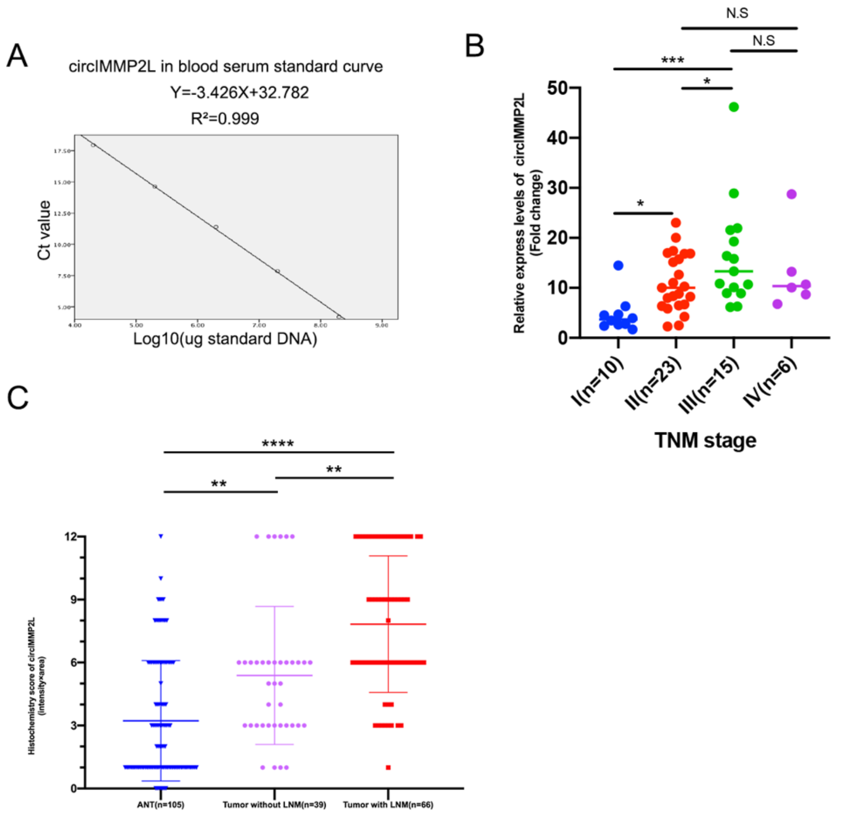

Figure S4 (A) The standard curve of circIMMP2L copy number per μg in blood plasma. (B) Scatter plots illustrating the qRT-PCR analysis of the expression fold change for circIMMP2L in 54 ESSC among TNM stage Ⅰ-Ⅳ. (normalized to ANT, ***p<0.001, *p<0.05 vs. stage Ⅰ/Ⅱ, two-tailed unpaired Student's t-test, n=54, N.S, no significant). (C) Histochemistry score of circIMMP2L among ANT, tumor without LNM, and tumor with LNM detected by In Situ Hybridization (ISH) assay in an ESCC tissue microarray (TMA) (***p*<0.01, *****p*<0.0001 vs. ANT or Tumor without LNM, two-tailed unpaired Student's *t* test, n=210).

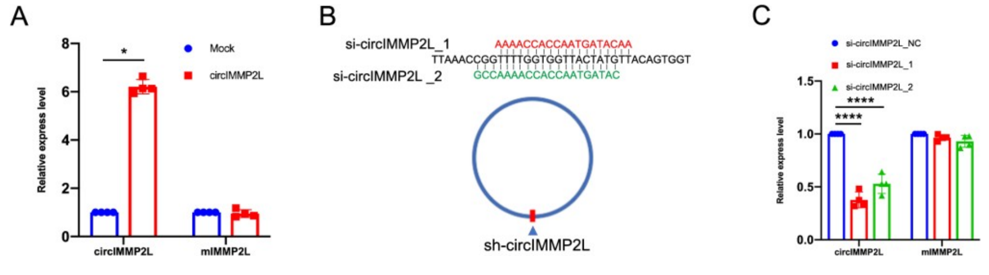

Figure S5 (A) The expression of circIMMP2L and mIMMP2L in Eca-109 cells

after transduction with circIMMP2L plasmid (*p <0.05 vs. Mock, two-tailed unpaired Student's t test, n=4). (B) The illustration showed the binding sites between circIMMP2L and sh-circIMMP2L. (C) The expression of circIMMP2L and mIMMP2L in TE-1 cells after transduction with sh-NC, sh-circIMMP2L_1, and sh-circIMMP2L_2(****p<0.0001 vs. sh-NC, two-tailed one-way ANOVA, n=4).

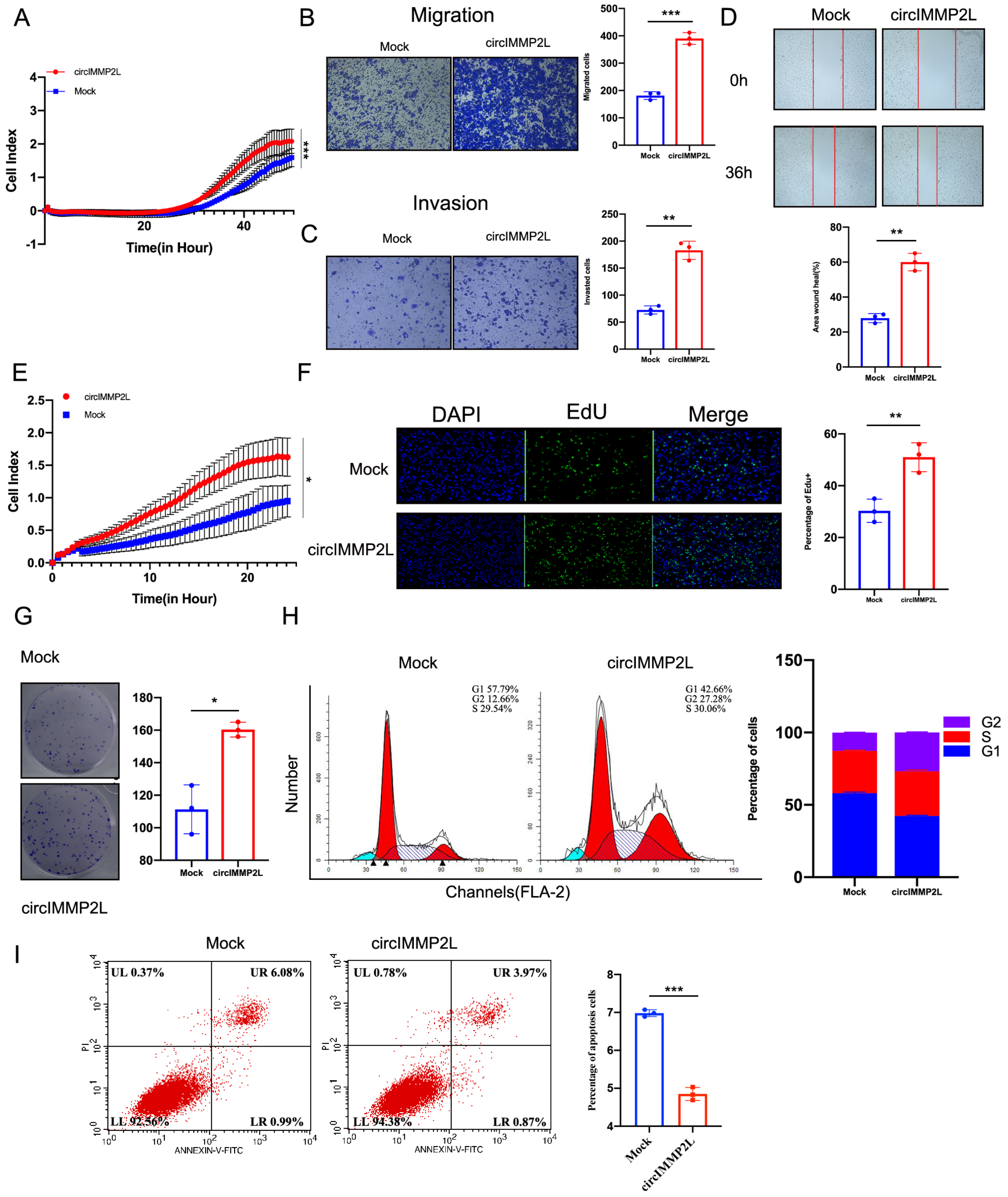

Figure S6 (A) Real-time cell analysis (RTCA) showed the invasion of Eca-109 cells stably transfected with empty vector (Mock) or circIMMP2L monitored by xCELLigence (***$p<0.001$ vs. Mock, multiple comparisons two-ANOVA, n=3). (B), (C) and (D) Transwell assay, matrigel assay and wound healing assay showed that overexpression of circIMMP2L promoted the migration and invasion ability of ESCC cells (**$p<0.01$, ***$p<0.001$ vs. Mock, two-tailed unpaired Student's *t* test, n=3). (E) RTCA showed the growth of Eca-109 cells stably transfected with empty vector (Mock) or circIMMP2L monitored by xCELLigence (*$p<0.05$ vs. Mock, multiple comparisons two-ANOVA, n=3). (F) and (G) EdU assay and colony formation assay showed that overexpression of circIMMP2L promoted the growth ability of ESCC cells. Left, representative images. Right, histograms of proliferation cell numbers. (H) and (I) Flow cytometric analysis of cell cycle progression and cell apoptosis in Eca-109 cells. The histograms indicate the percentages of cells (***$p<0.001$ vs. Mock, two- tailed unpaired Student's *t* test, n=3).

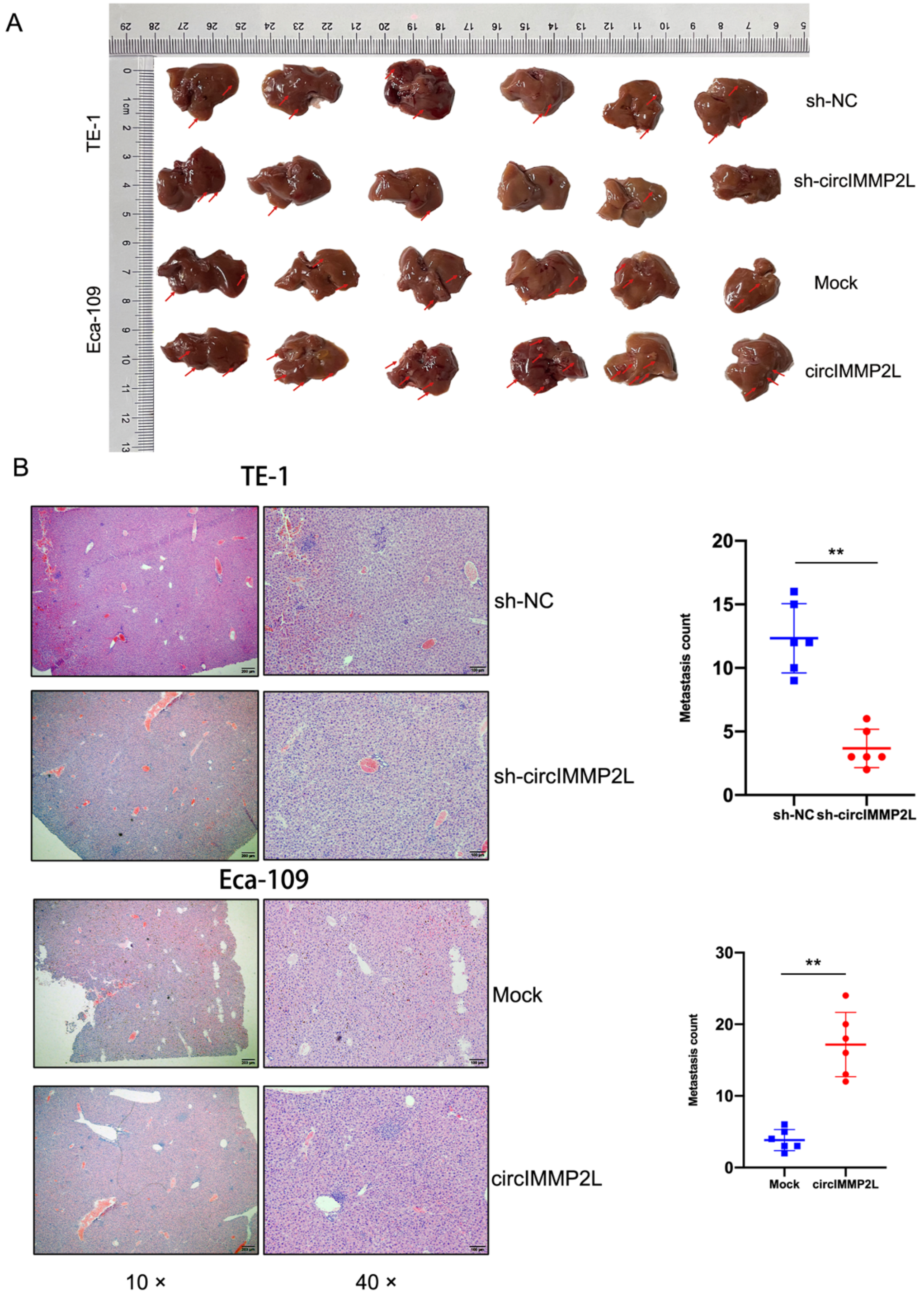

Figure S7 (A) Representative images showing the decreased or increased tumor metastasis formed in the livers of nude mice following vein tail injection of circIMMP2L-knockdown TE-1 cells or circIMMP2L-overexpressing Eca-109 cells. Metastases are indicated by arrows. (B) Left, representative HE staining of liver metastatic lesions. 10×, scar bar, 200μm. 40×, scar bar, 100μm. Right,

quantification of metastatic nodules forms in the liver of nude mice. (***$p<0.001$, ****$p<0.0001$vs. sh-NC, two-tailed unpaired Student's *t* test, n=6).

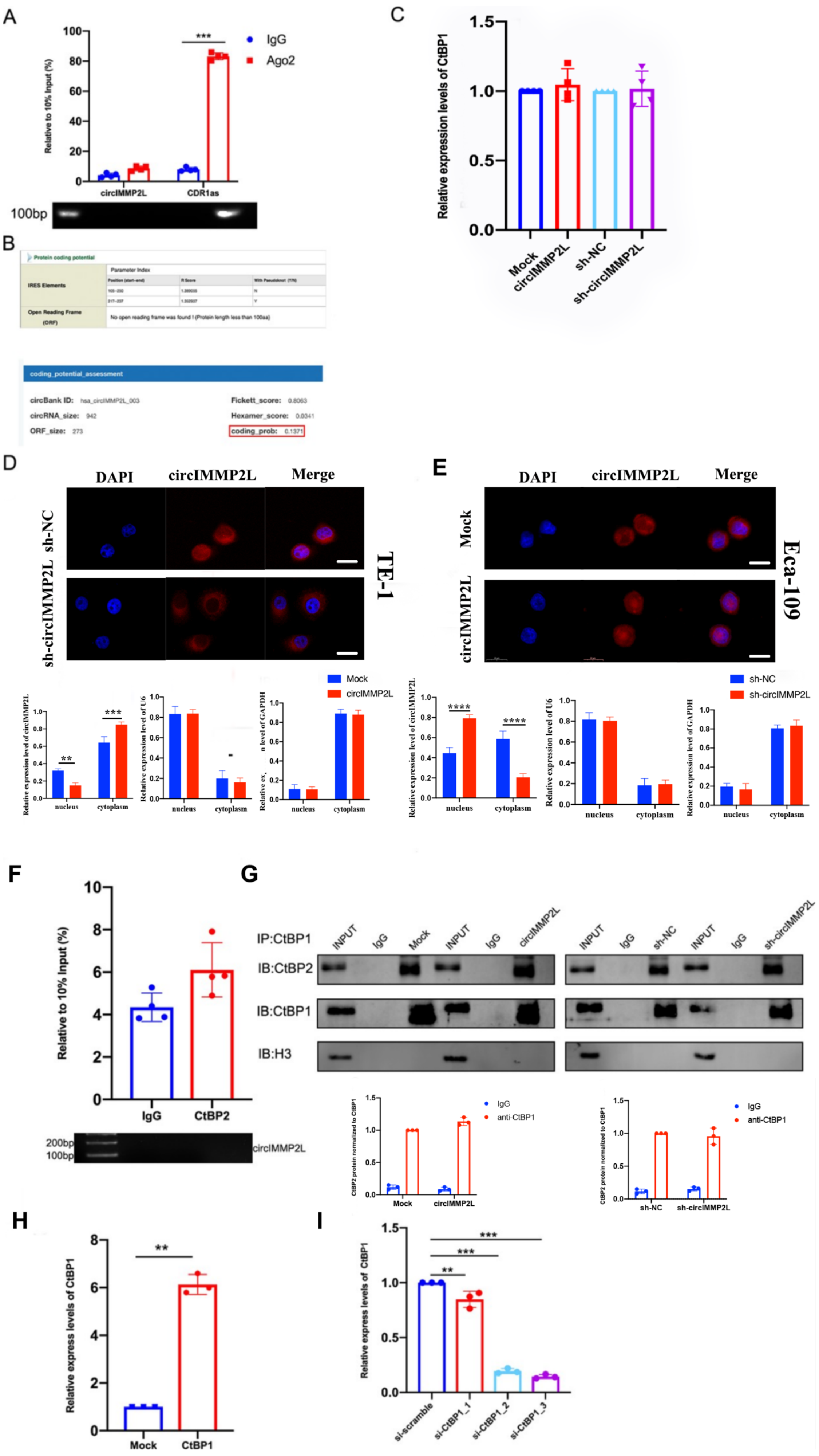

A
Relative to 10% Input (%)
IgG
Ago2
***
circIMMP2L
CDR1as
100bp
B
Protein coding potential
Parameter Index
IRES Elements
Open Reading Frame (ORF)
No open reading frame was found ! (Protein length less than 100aa)
coding_potential_assessment
circBank ID: hsa_circIMMP2L_003
circRNA_size: 942
ORF_size: 273
Fickett_score: 0.8063
Hexamer_score: 0.0341
coding_prob: 0.1371
C
Relative expression levels of CtBP1
Mock
circIMMP2L
sh-NC
sh-circIMMP2L
D
DAPI
circIMMP2L
Merge
sh-NC
sh-circIMMP2L
TE-1
E
Mock
circIMMP2L
Eca-109
Mock
circIMMP2L
sh-NC
sh-circIMMP2L
nucleus
cytoplasm
F
Relative to 10% Input (%)
IgG
CtBP2
200bp
100bp
circIMMP2L
G
IP:CtBP1
INPUT
IgG
Mock
circIMMP2L
sh-NC
sh-circIMMP2L
IB:CtBP2
IB:CtBP1
IB:H3
CtBP2 protein normalized to CtBP1
anti-CtBP1
H
Relative express levels of CtBP1
Mock
CtBP1
**
I
Relative express levels of CtBP1
si-scramble
si-CtBP1_1
si-CtBP1_2
si-CtBP1_3

Figure S8 (A) RIP assay showed the levels of circIMMP2L and CDR1as enriched by IgG and Ago2 anti-body (****p*<0.001 vs. IgG, two-tailed unpaired Student's *t* test, n=4).). (B) The illustration indicated that there was no ORF detected in circRNADb and the coding_prob predicted by circBank database. (C) The expression of CtBP1 in Eca-109 cells after transduction with circIMMP2L plasmid and in TE-1 cells after transduction with sh-circIMMP2L plasmid. (D) and (E) The FISH assay (Upper) and nuclear and cytoplasmic RNA extraction assay (lower) presented the distribution of circIMMP2L in nucleus and cytoplasm (****p*<0.001 ***p*<0.01 vs. Mock, sh-NC, multiple comparisons two-way ANOVA, n=3.) (F) RIP assay showed the levels of circIMMP2L enriched by IgG and CtBP2 anti-body. (G) Co-IP and western blot assays showed there were no differential interaction levels between CtBP1 and CtBP2 in Eca-109 cells stably transfected with vector(mock) or circIMMP2L and in TE-1 cells stably transfected with sh-NC or sh-circIMMP2L. (H) and (I) The expression of CtBP1 in TE-1 cells after transduction with circIMMP2L plasmid (***p* <0.01 vs. Mock, two-tailed unpaired Student's t test, n=3). The expression of CtBP1 in TE-1 cells after transduction with control (si-scramble) and CtBP1 siRNA (si-CtBP1_1, si- CtBP1_2, si-CtBP1_3) (***p*<0.01, ****p*<0.001 vs. si-scramble, two-tailed one-way ANOVA, n=3).

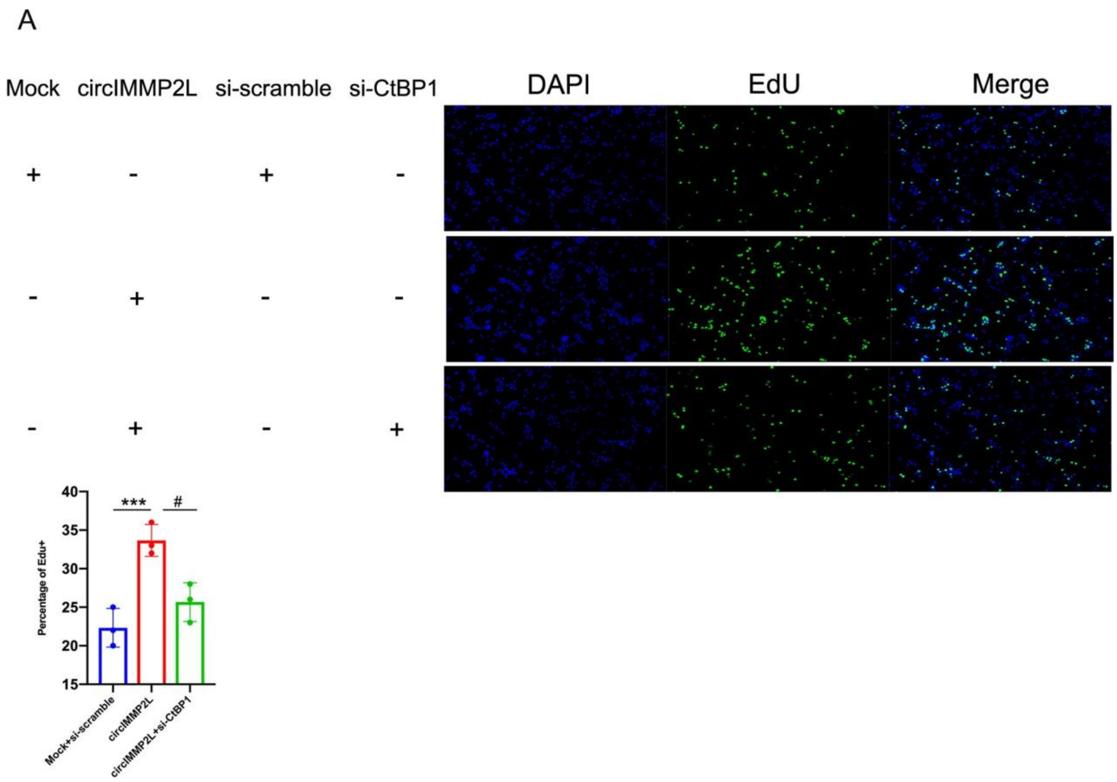

Figure S9 (A) EdU assays and quantification showed that overexpression of circIMMP2L cloud promote the proliferation ability of Eca-109 cells and that the growth could be blocked by the knockdown of CtBP1. (****p*<0.001 vs. Mock+si-scramble, #*p*<0.05 vs. circIMMP2L two-tailed unpaired Student's *t* test, n=3).

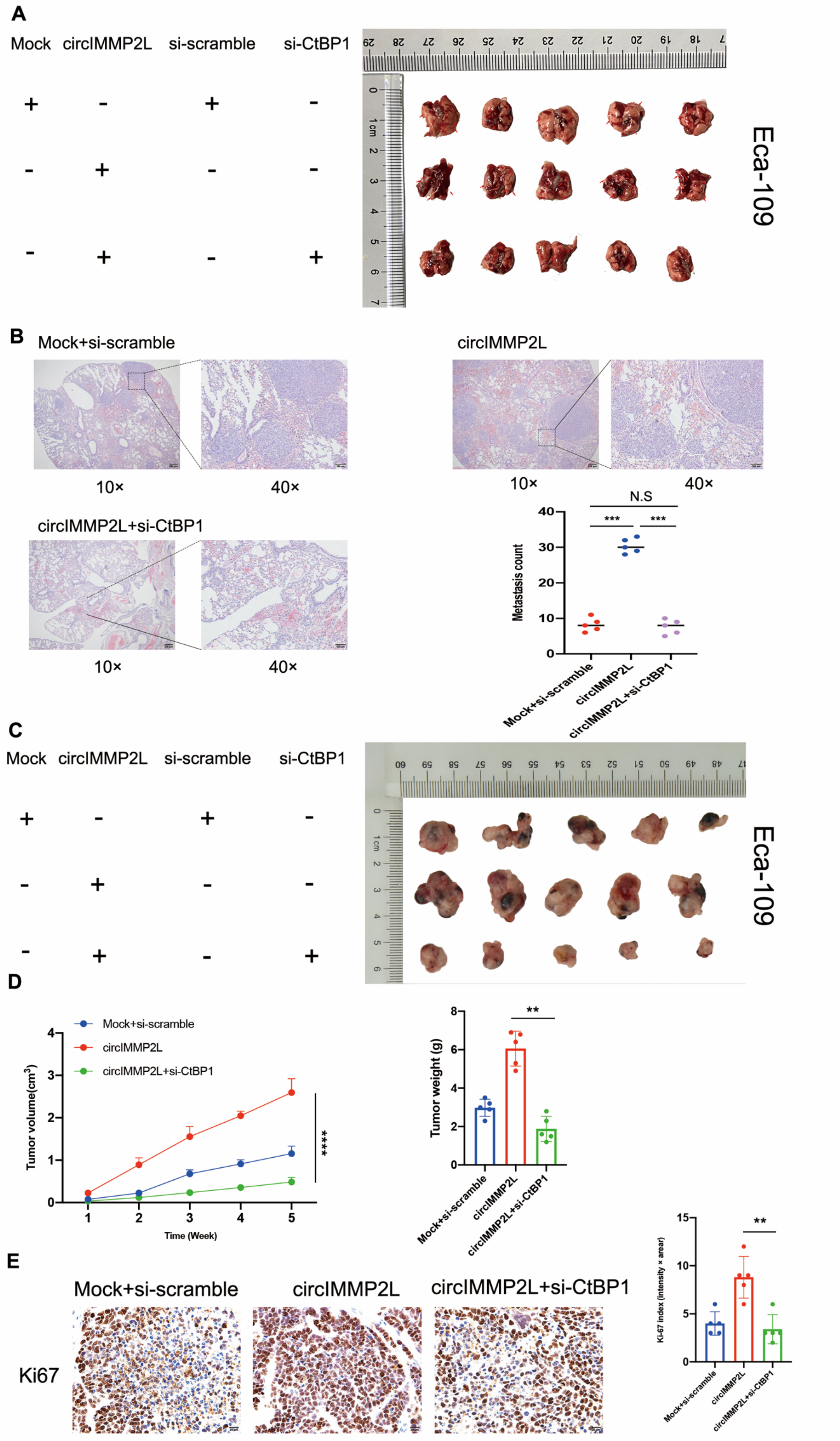

A
Mock
circIMMP2L
si-scramble
si-CtBP1
Eca-109
B
Mock+si-scramble
circIMMP2L
circIMMP2L+si-CtBP1
10×
40×
N.S
***
Metastasis count
C
Eca-109
D
Mock+si-scramble
circIMMP2L
circIMMP2L+si-CtBP1
Tumor volume(cm^3)
Time (Week)
****
Tumor weight (g)
**
E
Ki67
Ki-67 index (intensity × area)

Figure S10 (A) Representative images showing the decreased or increased tumor metastasis formed in the lungs of nude mice following vein tail injection of three kinds of treated Eca-109 cells. Metastases are indicated by arrows. (B) Representative HE staining of lung metastatic lesions (10×, scale bar, 200μm. 40×, scale bar, 100μm) and the quantification of metastatic nodules formed in the lungs of nude mice (***p<0.001, vs. Mock+si-scramble, circIMMP2L, two-tailed unpaired Student's t-test, n=5, N.S, not significant). (C) The representative image showing the subcutaneous xenograft tumor in nude mice derived from subcutaneous injection of three kinds of treated Eca-109 cells. (D) The volume (Left) and weight (Right) of subcutaneous xenograft tumors of Eca-109 isolated from nude mice (****p<0.0001 vs. circIMMP2L, multiple comparisons two-way ANOVA, n=6. **p<0.01 vs. circIMMP2L, two-tailed unpaired Student's t-test, n=5). (E) Representative images of Ki67 immunohistochemical staining within subcutaneous xenografts (**p<0.01 vs. circIMMP2L, two-tailed unpaired Student's t-test, n=5).

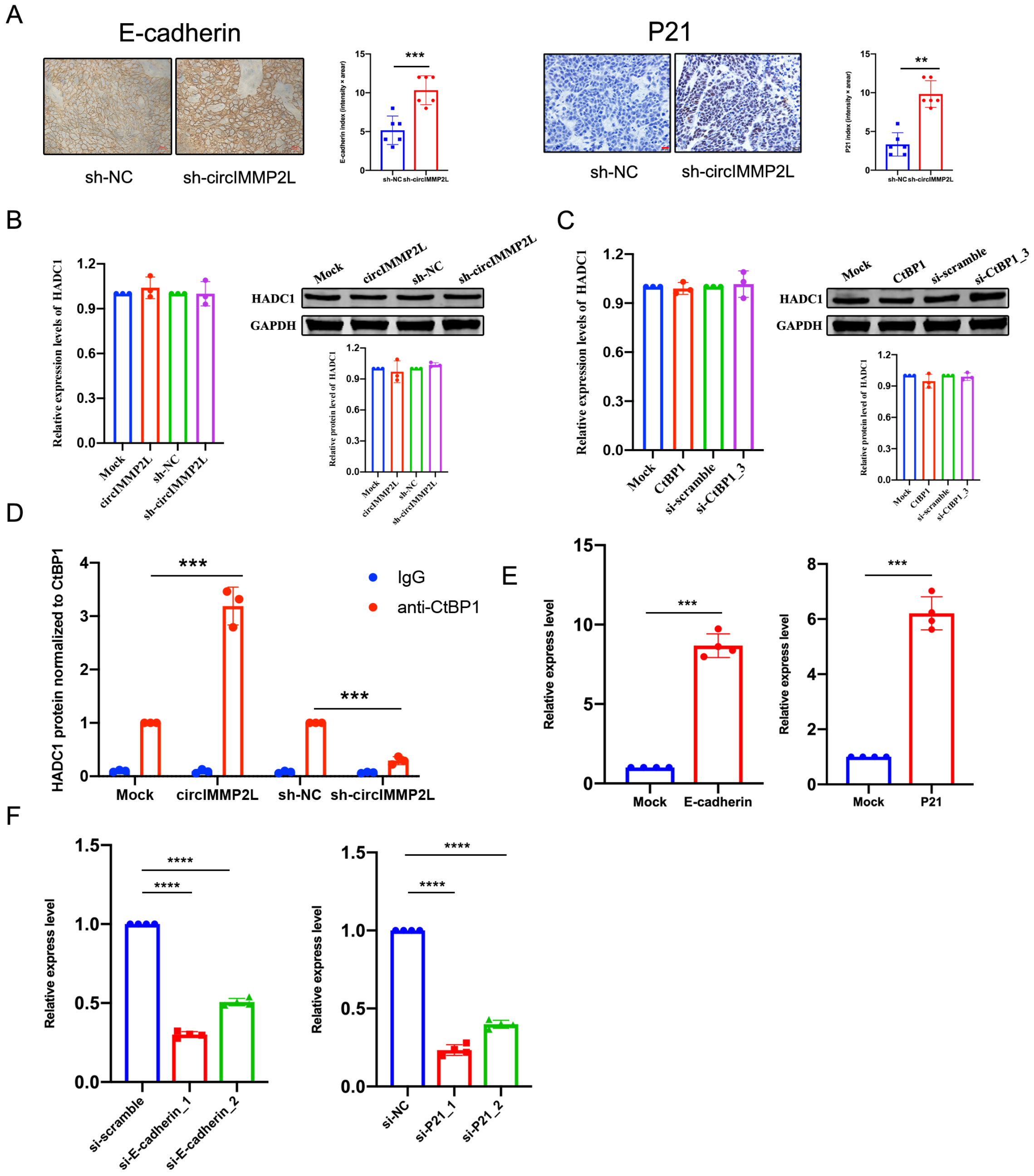

Figure S11 (A) Representative images and the quantification of E-cadherin and p21 immunohistochemical staining within subcutaneous xenografts derived from control (sh-NC) or circIMMP2L-knockdown TE-1 cells (**p<0.01 vs. sh-NC, two-tailed unpaired Student's *t* test, n=6). (B) The mRNA (Left) and protein (Right) expression level of HADC1 after over-expression and knockdown circIMMP2L. (C) The mRNA (Left) and protein (Right) expression level of HADC1 after over-expression and knockdown CtBP1. (D) The quantification of anti-CtBP1 IP assay (***p<0.001 vs. Mock, sh-NC, two-tailed unpaired Student's *t* test, n=3). (E) The expression of E-

cadherin and p21 in Eca-109 cells after transduction with E-cadherin and p21 plasmid (****p*<0.001 vs. Mock, two-tailed unpaired Student's *t* test, n=4). (F)The expression of E-cadherin and p21 in TE-1 cells after transduction with control (si-scramble), E-cadherin and siRNA (si -E-cadherin_1, si- E-cadherin _2, si-p21_1, si-p21_2) (*****p*<0.0001 vs. si-scramble, two-tailed one-way ANOVA, n=4).

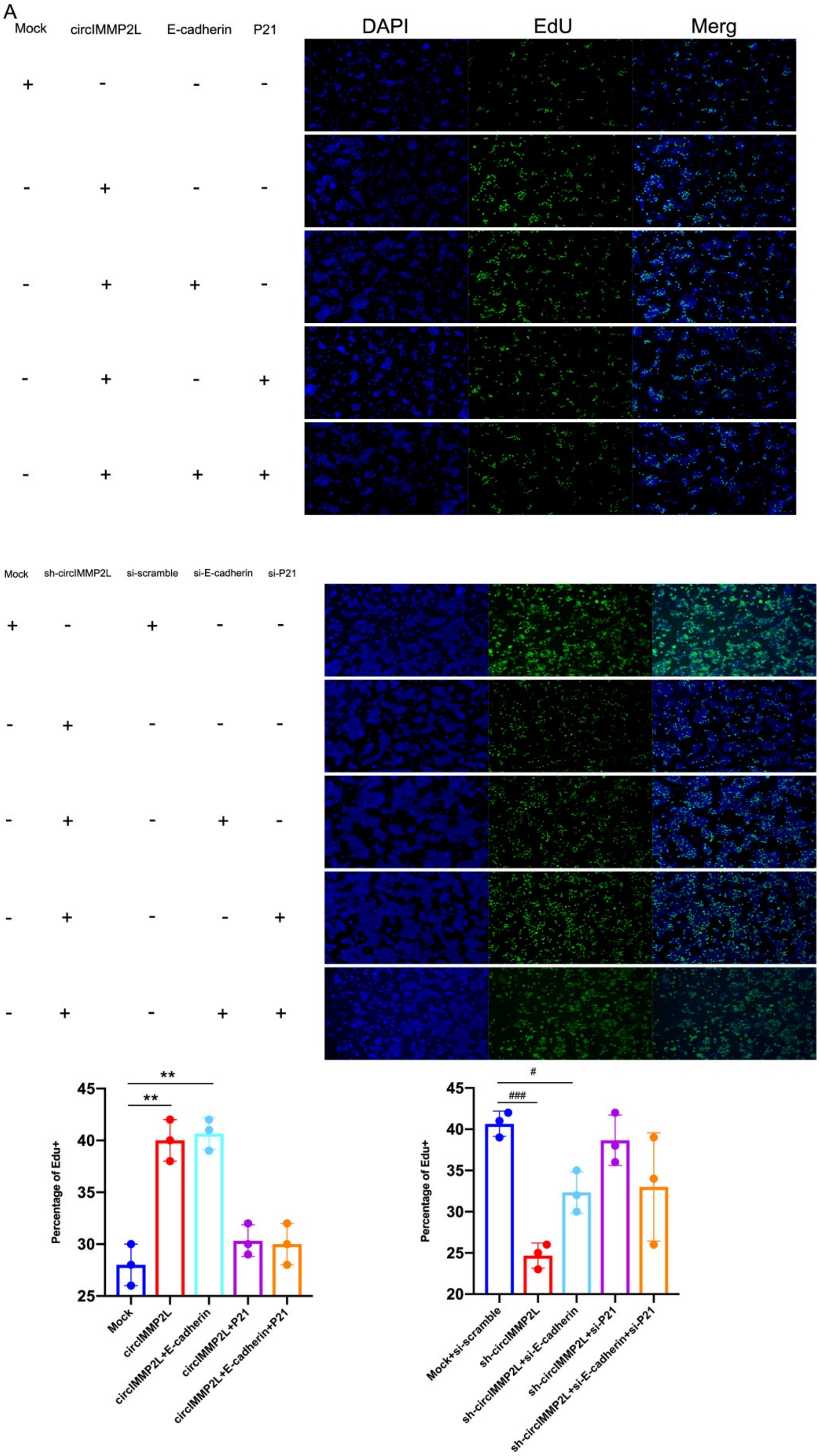
A
Mock
circIMMP2L
E-cadherin
P21
DAPI
EdU
Merg
Mock
sh-circIMMP2L
si-scramble
si-E-cadherin
si-P21
Percentage of Edu+
Mock
circIMMP2L
circIMMP2L+E-cadherin
circIMMP2L+P21
circIMMP2L+E-cadherin+P21
Mock+si-scramble
sh-circIMMP2L
sh-circIMMP2L+si-E-cadherin
sh-circIMMP2L+si-P21
sh-circIMMP2L+si-E-cadherin+si-P21

Figure S12 (A) EdU assays and quantifications showed that overexpression of circIMMP2L cloud promote the proliferation ability of Eca-109 cells and that the growth could be blocked by p21-overexpression. Knockdown of p21 could rescue the proliferation ability suppression of circIMMP2L-knockdown in TE-1 cells (**p<0.01 vs. Mock, $^{###}p$<0.001, $^{\#}p$<0.05 vs. Mock+si-scramble two-tailed unpaired Student's t test, n=3
