# Supplementary material for: CircIMMP2L promotes esophageal squamous cell carcinoma malignant progression via CtBP1 nuclear retention dependent epigenetic modification
Source: Clin Transl Med. 2021 Sep 26;11(9):e519. doi: 10.1002/ctm2.519 (PMC8473481; doi:10.1002/ctm2.519)
Supplement: Supplementary file 2 — Supporting Information [file CTM2-11-e519-s003.docx]

**Table S1 Clinical Characteristics of 105 ESCC Patients in this study**

|  | **Cohort1** | **Cohort2** | **Cohort3** |
| --- | --- | --- | --- |
| **All cases** | **3** | **54** | **48** |
| **Age** | | | |
| **≥60** | **1** | **31** | **8** |
| **<60** | **2** | **23** | **40** |
| **Gender** | | | |
| **Male** | **1** | **43** | **35** |
| **Female** | **2** | **11** | **13** |
| **Lymph node metastasis** | | | |
| **positive** | **3** | **31** | **24** |
| **negative** | **0** | **23** | **24** |
| **Tumor diameter** | | | |
| **≥5cm** | **0** | **26** | **－** |
| **<5cm** | **3** | **28** | **－** |
| **TNM stage** | | | |
| **I** | **0** | **10** | **24** |
| **II and III** | **3** | **44** | **24** |
| **Pathological grading** | | | |
| **G1** | **0** | **12** | **28** |
| **G2-G4** | **3** | **42** | **20** |

**Table S2 Primers, Probes, and RNA sequences used in this study**

| **Primers for qRT-PCR** | |
| --- | --- |
| **Primer Name** | **Sequence (5’-3’)** |
| **circIMMP2L-F** | **AGTCATCTGATGTGGTGCTTT** |
| **circIMMP2L-R** | **CCTCGGTGAGAAGGGGTAAG** |
| **circCNTNAP3-F** | **AATGTGGTGGTGGACGATGAC** |
| **circCNTNAP3-F** | **GCAGAGCACTTTGTCCATCAA** |
| **circLIFR-F** | **GGAACGACAGGGGTTCAGTT** |
| **circLIFR-R** | **CCAGGATGGTCGTTTCAAACAT** |
| **circSHC3-F** | **CTGTGTGGAGTGGAAGATGGA** |
| **circSHC3-R** | **GGAGTCCATTTTCAAGAGGGC** |
| **IMMP2L-F** | **CCGGTATGTCAAAGTCCCCC** |
| **IMMP2L-R** | **GAAGTCCTAGGGAAACCGGC** |
| **GAPDH-F** | **GGAGCGAGATCCCTCCAAAAT** |
| **GAPDH-R** | **GGCTGTTGTCATACTTCTCATGG** |
| **U6-F** | **CTCGCTTCGGCAGCACA** |
| **U6-R** | **AACGCTTCACGAATTTGCG** |
| **FUS-F** | **ATGGCCTCAAACGATTATACCCA** |
| **FUS-R** | **GTAACTCTGCTGTCCGTAGGG** |
| **CDR1as-F** | **TCAACTGGCTCAATATCCATGTC** |
| **CDR1as-R** | **ACCTTGACACAGGTGCCAT** |
| **CtBP1-F** | **AAAGCCCTCCGCATCATCG** |
| **CtBP1-R** | **AGACGGCAATGCCTAAATCCC** |
| **E-cadherin-F** | **ATTTTTCCCTCGACACCCGAT** |
| **E-cadherin-R** | **TCCCAGGCGTAGACCAAGA** |
| **P21-F** | **TGTCCGTCAGAACCCATGC** |
| **P21-R** | **AAAGTCGAAGTTCCATCGCTC** |
| **PERP-F** | **CTTCACCCTTCATGCCAACC** |
| **PERP-R** | **GCCAATCAGGATAATCGTGGCT** |
| **P16-F** | **GATCCAGGTGGGTAGAAGGTC** |
| **P16-R** | **CCCCTGCAAACTTCGTCCT** |
| **PTEN-F** | **TTTGAAGACCATAACCCACCAC** |
| **PTEN-R** | **ATTACACCAGTTCGTCCCTTTC** |
| **Bax-F** | **CCCGAGAGGTCTTTTTCCGAG** |
| **Bax-R** | **CCAGCCCATGATGGTTCTGAT** |
| **BIK-F** | **GACCTGGACCCTATGGAGGAC** |
| **BIK-R** | **CCTCAGTCTGGTCGTAGATGA** |
| **BBC3-F** | **GCCAGATTTGTGAGACAAGAGG** |
| **BBC3-R** | **CAGGCACCTAATTGGGCTC** |
| **E-cadherin Promotor-F** | **CAGCTACTAGAGAGGCTGGGGCCAG** |
| **E-cadherin Promotor-R** | **CGTACCGCTGATTGGCTGAGGGTTC** |
| **P21 Promotor-F** | **GCTGGGCAGCCAGGAGCCTG** |
| **P21 Promotor-R** | **CTGCTCACACCTCAGCTGGC** |
| **Hsa_circ_0081096-F** | **CGGTATTCCTGGCCCTGTT** |
| **Hsa_circ_0081096-R** | **GAAGACCACGAGAACCAGGA** |
| **NA#1-F** | **CTTACTGGTGCCAAGGGTG** |
| **NA#1-R** | **CTTACTGGTGCCAAGGGTG** |
| **Hsa_circ_0024251-F** | **GGTGAAGCGGCATAATGTGT** |
| **Hsa_circ_0024251-R** | **AGCAAGACCAAGAGACAGGG** |
| **Hsa_circ_0041551-F** | **GGGCAAAAGCAATGAGTGGA** |
| **Hsa_circ_0041551-R** | **GCACATTCGTTGCAGGGTAT** |
| **NA#2-F** | **CGCTTGCATACCACCAATCA** |
| **NA#2-R** | **GCTGGTAAACGATCTCCCCT** |
| **Hsa_circ_0084789-F** | **TGGCAACCCTGTTCACCTAA** |
| **Hsa_circ_0084789-R** | **GCATTAGGGCAGTGGTGTTC** |
| **Hsa_circ_0002078-F** | **CGGCTTCTCCTGTCTGACAT** |
| **Hsa_circ_0002078-R** | **GCCCAGTCTCTTTCTCAAGC** |
| **Hsa_circ_0015879-F** | **CCTCAGACAGCAAGGTTTCG** |
| **Hsa_circ_0015879-R** | **GCATGCCCAGTCTCTTTCTC** |
| **Hsa_circ_0038072-F** | **TCGATAGGAACATGCTGGGA** |
| **Hsa_circ_0038072-R** | **TGAGCCCTGAGTAGTTACGC** |
| **Hsa_circ_0026428-F** | **ACTGTCCAAGCAAGTTAGGC** |
| **Hsa_circ_0026428-R** | **GTAGTTACGCATGGTCTGGC** |
| **Hsa_circ_0004846-F** | **ACTGGCAGGACAAAAGCATG** |
| **Hsa_circ_0004846-R** | **TCCTTGGATTCCTGTTGCCA** |
| **Hsa_circ_0005568-F** | **GGTGTGAGAATGGCCATGTG** |
| **Hsa_circ_0005568-R** | **TGGATTCCTGTTGCCATTGG** |
| **Hsa_circ_0000217-F** | **GGCCTGACCCATCTGAAGTT** |
| **Hsa_circ_0000217-R** | **CCATGATGCACACAGAGGAC** |
| **Hsa_circ_0002474-F** | **AGGATCCTCGGTTCAATGCA** |
| **Hsa_circ_0002474-R** | **ACAGGGATGGTGTGAACTCT** |
| **Hsa_circ_0000325-F** | **TGGGAGTGGTAGGATGAAACA** |
| **Hsa_circ_0000325-R** | **AAAGCATTGCCCTTCTATTGGT** |
| **Hsa_circ_0070805-F** | **CAGCAGCAGAGGAAGAAACA** |
| **Hsa_circ_0070805-R** | **GGTGTGGCCTCTGATACCTT** |
| **Hsa_circ_0008144-F** | **GTCACATTGGAGGTTCTGTCG** |
| **Hsa_circ_0008144-R** | **ATGTCCCACCGTTTCCTGAT** |
| **Hsa_circ_0006867-F** | **TGCCCACCAACTTCAGAGAT** |
| **Hsa_circ_0006867-R** | **CTCCAGGGCTGTATTTTGCA** |
| **Hsa_circ_0087104-F** | **GGACGATGACACAGCTGTTC** |
| **Hsa_circ_0087104-R** | **GCAGAGCACTTTGTCCATCA** |

| **Pull-down probe** | |
| --- | --- |
| **circIMMP2L** | **Biotin-GCCAAAACCACCAATGATACAATGT** |
| **scramble** | **Biotin-GTGTAACACGTCTATACGCCCA** |

| **FISH probe** | |
| --- | --- |
| **circIMMP2L** | **Cy3-GCCAAAACCACCAATGATACAATGT** |

| **ISH probe** | |
| --- | --- |
| **circIMMP2L** | **Biotin-GCCAAAACCACCAATGATACAATGT** |

| **siRNAs** | |
| --- | --- |
| **Oligo set** | **Target sequences** |
| **si-circIMMP2L_1** | **TTGTATCATTGGTGGTTTT** |
| **si-circIMMP2L_2** | **ATTGGTGGTTTTGGCCAAA** |
| **si-FUS_1** | **GAGTGGAGGTTATGGTCAA** |
| **si-FUS_2** | **GACCAAAAAUAAAAACAAA** |
| **si-CtBP1_1** | **CTTGGGCATCATCGGACTT** |
| **si-CtBP1_2** | **GCTTCAACGTGCTCTTCTA** |
| **si-CtBP1_3** | **GTGTCAACAAGGACCATCT** |
| **si-E-cadherin_1** | **CTCCGTTTCTGGAATCCAA** |
| **si-E-cadherin_2** | **GGAGATTAATCCGGACACT** |
| **si-P21_1** | **GATGGAACTTCGACTTTGT** |
| **si-P21_2** | **AGACCATGTGGACCTGTCA** |

| **Oligo for shRNAs construction** | |
| --- | --- |
| **pGFP-u6- sh-circIMMP2L** | **CACCGAAAACCACCAATGATACAA TTCAAGAGA TTGTATCATTGGTGGTTTTTTTTTTG (sense)** |
|  | **GATCCAAAAAAAAAACCACCAATGATACAATCTC**  **TTGAATTGTATCATTGGTGGTTTTC (antisense)** |

| **Oligo for plasmid construction** | |
| --- | --- |
| **Oligo set** | **The sequence** |
| **pcDNA3.1-circIMMP2L** | **TAGTTAAGCTTGGTACCGAGCTCGGATCCAaag**  **Tgctgagattacagg (sense)** |
|  | **ACTGTGCTGGATATCTGCAGAATTCtgctgggat**  **Tacaggtgtgagcta (antisense)** |
| **Flag-CtBP1-full length** | **AAAGCTAGCGCCACCATGGATTACAAGGATGACGA**  **CGATAAG ATGGGCAGCTCGCACTTGCTCA (sense)** |
|  | **AAA GAATTC TCA CAACTGGTCA CTGGCGTGGT CT (antisense)** |
| **Flag-CtBP1-doaminA** | **AAAGCTAGCGCCACCATGGATTACAAGGATGACGACGTAAG ATGGGCAGCTCGCACTTGCTCA (sense)** |
|  | **AAAGAATTCTCAAGACGCCGCG GGCACGTTGC AG (antisense)** |
| **Flag-CtBP1-doaminB** | **AAAGCTAGCGCCACCATGGATTACAAGGATGACGACGATAAGGTGGAGGAGACGGCCGACTCG** |
|  | **AAA GAATTC TCA CCATGCAGCA TGGGGGGTGC AG** |
| **Flag-CtBP1-doaminC** | **AAAGCTAGCGCCACCATGGATTACAAGGATGACGACGA TAAG TACAGCGAGCAGGCATCCATCG (sense)** |
|  | **AAAGAATTCTCACAACTGGTCACTGGCGTGGTCT (antisense)** |
| **Flag-CtBP1-doaminB+C** | **AAAGCTAGCGCCACCATGGATTACAAGGATGACGACGA TAAG GTGGAGGAGACGGCCGACTCG (sense)** |
|  | **AAAGAATTC TCACAACTGGTCACTGGCGTGGTCT (antisense)** |
| **PC-h-E-cadherin** | **TAGTTAAGCTTGGTACCGAGCTCGGATCCGCCAC**  **CATGGGCCCTT GGAG (sense)** |
|  | **CACTGTGCTGGATATCTGCAGAATTCCTATTTGTC**  **GTCATCATCCTT AT (antisense)** |

**Table S3 The antibodies used in this study**

| **Antibody** | **Supplier** | **Catalogue number** | **Host** |
| --- | --- | --- | --- |
| **CtBP1** | **CST** | **#8684** | **R** |
| **GAPDH** | **CST** | **#5174** | **R** |
| **Histon H3** | **CST** | **#4499** | **R** |
| **IGF2BP1** | **Abcam** | **ab82968** | **R** |
| **PKM2** | **Abcam** | **#4053** | **R** |
| **CtBP2** | **CST** | **#13256** | **R** |
| **Ago2** | **Abcam** | **ab32381** | **R** |
| **E-cadherin** | **CST** | **#14472** | **M** |
| **P21** | **CST** | **#2947** | **M** |
| **HDAC1** | **Abcam** | **ab213701** | **R** |
| **CD31** | **proteintech** | **11265-1-AP** | **R** |
| **H3K9AC** | **CST** | **#9649** | **R** |
| **Ki67** | **proteintech** | **27309-1-AP** | **R** |
| **IgG** | **CST** | **#3900** | **R** |

**CtBP1: CST: Cell Signal Technology; R: Rabbit; M: Mouse;**

**Table S6 The full length of circIMMP2L measured by Sanger sequencing**


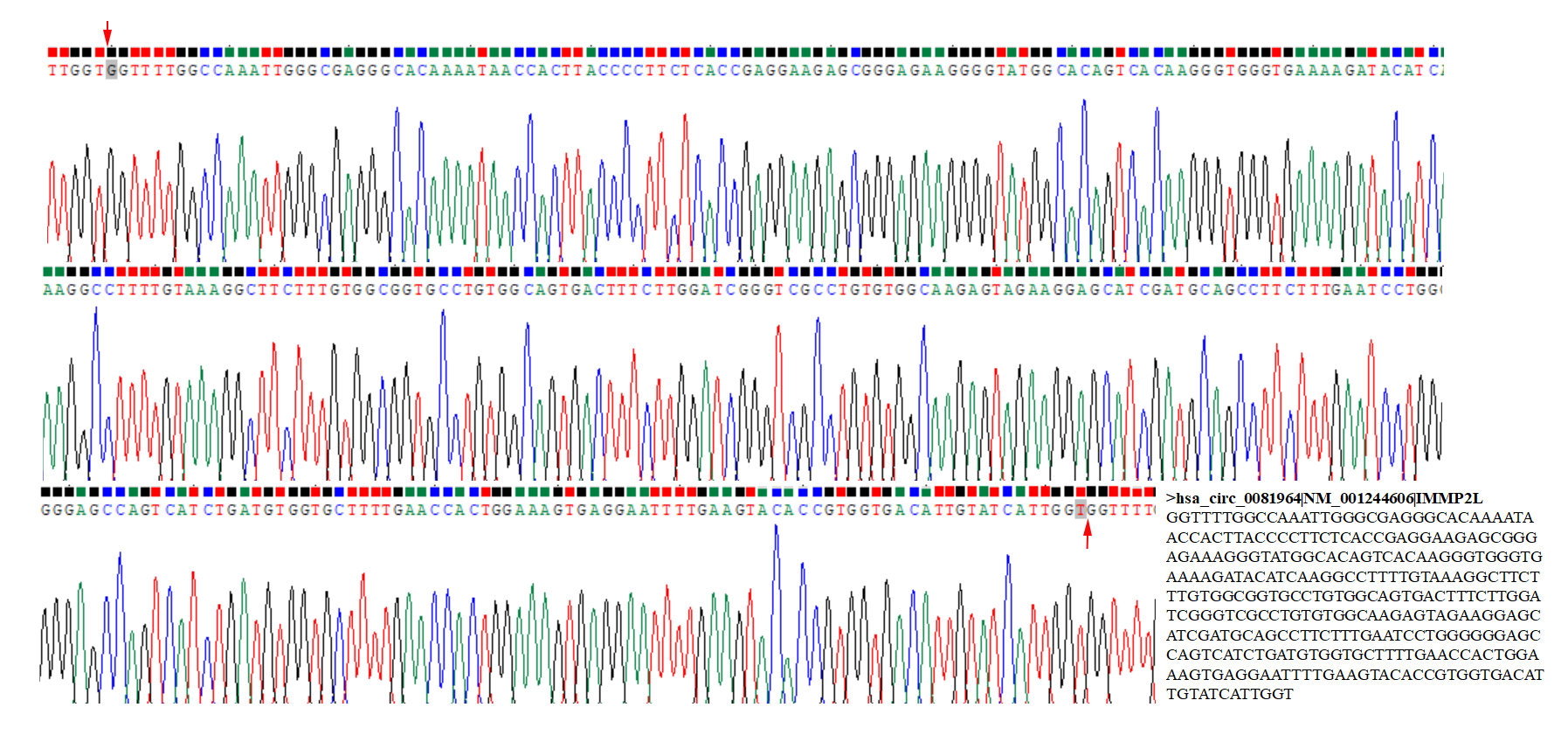


**Table S7 Univariate and Multivariable analysis of variables for overall survival (OS)**

| **Variables** | **Univariate analysis** | | | **Multivariable analysis** | | |
| --- | --- | --- | --- | --- | --- | --- |
|  | **HR** | **95%CI** | **p value** | **HR** | **95%CI** | **p value** |
| **Age (>60)** | **1.734** | **1.029-2.923** | **0.039** | **2.025** | **1.262-3.529** | **0.013** |
| **Gender (male)** | **0.974** | **0.619-1.533** | **0.910** |  |  |  |
| **Lymph node metastasis (positive)** | **6.545** | **3.804-11.262** | **<0.0001** | **2.611** | **1.420-4.801** | **0.002** |
| **Tumor diameter (≥5cm)** | **1.884** | **1.210-2.934** | **0.005** | **1.817** | **1.128-2.928** | **0.014** |
| **Pathological grading (G2-G4)** | **1.677** | **1.043-2.699** | **0.033** | **1.129** | **0.667-1.909** | **0.652** |
| **circIMMP2L (high)** | **5.194** | **3.127-8.629** | **<0.0001** | **4.272** | **2.257-8.086** | **0.001** |
